# Soluble immune checkpoints: implications for cancer prognosis and response to immune checkpoint therapy and conventional therapies

**DOI:** 10.1186/s13046-024-03074-z

**Published:** 2024-05-31

**Authors:** Stephanie C. Pitts, Jeffrey Schlom, Renee N. Donahue

**Affiliations:** Center for Immuno-Oncology, Center for Cancer Research, National Cancer Institute, National Institutes of Health, Bethesda, MD USA

**Keywords:** Peripheral immunome, Blood analyses, Soluble immune checkpoints, Biomarkers, Immune checkpoint inhibitors, Conventional cancer therapies

## Abstract

Longitudinal sampling of tumor tissue from patients with solid cancers, aside from melanoma and a few other cases, is often unfeasible, and thus may not capture the plasticity of interactions between the tumor and immune system under selective pressure of a given therapy. Peripheral blood analyses provide salient information about the human peripheral immunome while offering technical and practical advantages over traditional tumor biopsies, and should be utilized where possible alongside interrogation of the tumor. Some common blood-based biomarkers used to study the immune response include immune cell subsets, circulating tumor DNA, and protein analytes such as cytokines. With the recent explosion of immune checkpoint inhibitors (ICI) as a modality of treatment in multiple cancer types, soluble immune checkpoints have become a relevant area of investigation for peripheral immune-based biomarkers. However, the exact functions of soluble immune checkpoints and their roles in cancer for the most part remain unclear. This review discusses current literature on the production, function, and expression of nine soluble immune checkpoints – sPD-L1, sPD-1, sCTLA4, sCD80, sTIM3, sLAG3, sB7-H3, sBTLA, and sHVEM – in patients with solid tumors, and explores their role as biomarkers of response to ICI as well as to conventional therapies (chemotherapy, radiotherapy, targeted therapy, and surgery) in cancer patients.

**Supplementary Information:**

The online version contains supplementary material available at 10.1186/s13046-024-03074-z.

## Introduction

Peripheral blood analyses provide salient information about the human peripheral immunome while offering technical and practical advantages over tumor biopsies [1–5]. Tumor biopsies have been traditionally analyzed for protein expression and/or tumor mutational burden (TMB) to identify biomarkers of treatment response [1]. However, tumor specimens often do not account for intratumoral heterogeneity or heterogeneity between the primary tumor and metastases, and are difficult to obtain at multiple time points to assess changes over time [1, 2, 4]. Practically, tumor biopsies are expensive and can cause both treatment delays and the potential risk of adverse events [2]. On the other hand, obtaining blood from patients is non-invasive, low-risk, and can be performed repeatedly over multiple time points [1–5]. In recent years, an effort has been made to develop blood-based biomarkers in cancer patients to study the systemic effects of a given therapy on the immune system; when possible, these studies should be used to complement methods that directly interrogate the tumor and tumor microenvironment. Some common blood-based biomarkers include immune cell subsets, the neutrophil-to-lymphocyte ratio (NLR), circulating tumor DNA, and protein analytes such as cytokines [1–5]. With the introduction of immune checkpoint inhibitors (ICI) as a widespread modality of cancer treatment, soluble immune checkpoints have become a relevant area of investigation for potentially identifying peripheral immune-based biomarkers.

**Fig. 1:**
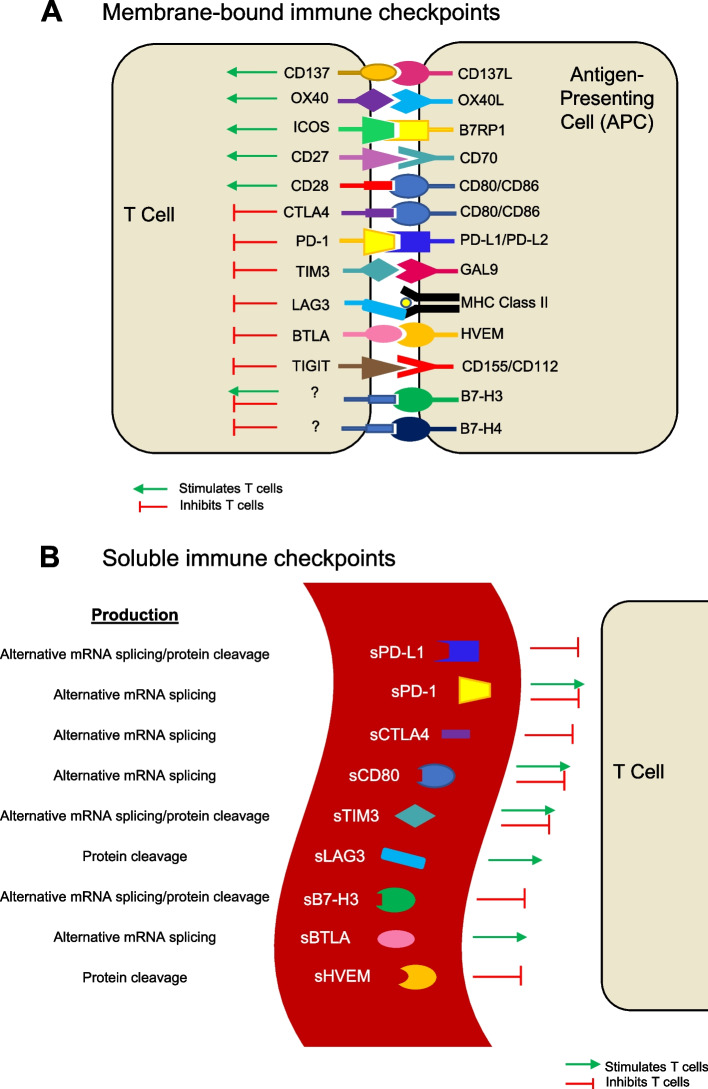
Membrane-bound (**A**) and soluble (**B**) immune checkpoints. Soluble immune checkpoints are produced through cleavage of the membrane-bound immune checkpoint proteins and/or alternative splicing of mRNA. ICOS, inducible T cell co-stimulator; B7RP1, B7-related protein 1; CTLA4, cytotoxic T-lymphocyte-associated antigen 4; PD-1, programmed cell death protein 1; PD-L1, programmed cell death-ligand 1; PD-L2, programmed cell death-ligand 2; TIM3, T cell immunoglobulin and mucin domain-containing protein 3; GAL9, galectin 9; LAG3, lymphocyte activation gene 3; MHC, major histocompatibility complex; BTLA, B and T lymphocyte attenuator; HVEM, herpesvirus entry mediator; TIGIT, T cell immunoglobulin and ITIM domain

Immune checkpoints are stimulatory or inhibitory signaling molecules that regulate T cell response upon antigen presentation [6–10]. The ligand is often found on the antigen-presenting cell (APC), while its corresponding receptor is typically located on the T cell [9]. Stimulatory molecules include CD137, CD137L, OX40, OX40L, CD28, CD86, CD80, inducible T cell co-stimulator (ICOS), B7-related protein 1 (B7RP1), CD27, and CD70, while inhibitory molecules include programmed cell death protein 1 (PD-1), programmed cell death-ligand 1 (PD-L1), programmed cell death-ligand 2 (PD-L2), cytotoxic T-lymphocyte-associated antigen 4 (CTLA4), CD86, CD80, T cell immunoglobulin and mucin domain-containing protein 3 (TIM3), galectin 9 (GAL9), lymphocyte activation gene 3 (LAG3), B and T lymphocyte attenuator (BTLA), herpesvirus entry mediator (HVEM), T cell immunoglobulin and ITIM domain (TIGIT), B7-H3, and B7-H4 (Fig. 1A) [9, 11]. Blocking inhibitory immune checkpoints can enable T cell activation upon recognition of a tumor antigen, thus harnessing the power of the immune system to destroy cancer cells [6–9]. The CTLA4 inhibitor ipilimumab was the first ICI to obtain FDA approval in 2011 [9, 12]. Since then, anti-PD-L1, anti-PD-1, and anti-CTLA4 antibodies have become widespread as cancer therapeutic agents and are utilized across multiple cancer types [6]. Many studies are currently investigating inhibitors of other inhibitory immune checkpoints, such as LAG3, TIM3, and TIGIT, and agonists of stimulatory immune checkpoints, such as CD137, OX40 and ICOS [6–8, 11, 13]. Despite the promise and widespread use of ICI, many patients are resistant to this modality of treatment [14, 15]. The mechanisms of resistance are unclear, and it is unknown which patients will derive clinical benefit [14, 15]. It is therefore imperative to identify biomarkers that can serve as predictors of clinical response in patients with solid tumors receiving ICI.

Immune checkpoints can exist in two forms – membrane-bound and soluble [10, 16, 17]. Membrane-bound immune checkpoints are found on both cell membranes and exosomal membranes [10, 18, 19], while soluble immune checkpoints are produced through the alternative splicing of mRNA or the cleavage of membrane-bound immune checkpoint proteins (Fig. 1B) [10, 16, 17]. Soluble immune checkpoints have been observed in bodily fluids such as blood (plasma and serum) [10, 16, 20–22], urine [21], cerebrospinal fluid [20, 22], and peritoneal fluid [23, 24], and their levels can be measured quantitatively through methods such as ELISAs or multiplexed assays [10, 16, 17]. However, the exact functions of soluble immune checkpoints and their roles in normal biology and diseased states such as cancer remain unclear [10, 16, 17].

This review will discuss nine soluble immune checkpoints measured in plasma or serum and summarize what is known in terms of their production, function, expression, and association with tumor stage in patients with solid tumors other than melanoma. The potential role of these soluble immune checkpoints to serve as biomarkers of clinical response in patients with cancer, both prior to and during treatment with ICI or conventional therapies (e.g., chemotherapy, radiotherapy, targeted therapy, surgery, and combinations of these treatment modalities), will be discussed. To our knowledge, this is the first review to comprehensively compare the biomarker potential of multiple soluble immune checkpoints, at time points both before and during therapy, in patients with solid tumors other than melanoma receiving different treatment modalities.

## Levels of soluble immune checkpoints as indicators of clinical response to ICI

Several studies have reported that baseline, or pre-treatment, levels of soluble immune checkpoints can indicate clinical response to ICI. In addition, circulating levels of soluble immune checkpoints can change upon treatment with ICI, and in some cases, the magnitude of this change associates with clinical response.

### sPD-L1

#### Structure and function of membrane-bound PD-L1

Programmed cell death-ligand 1 (PD-L1) is expressed on APCs (such as macrophages and dendritic cells), T cells, and tumor cells [25–28]. It is a transmembrane glycoprotein that consists of immunoglobulin C-like (IgC) and immunoglobulin variable-type (IgV-type) extracellular domains, a transmembrane domain, and a cytoplasmic domain [25, 28]. The binding of PD-L1 to PD-1 induces the intracellular phosphorylation of PD-1; phosphorylated PD-1 recruits Src homology region 2 domain-containing phosphatase-1/2 (SHP-1/2), which downregulates downstream pathways such as the PI3K-AKT-mTOR and RAS-MEK-ERK pathways [29–32]. The downregulation of these pathways suppresses T cell growth, survival, and proliferation [32–34]; thus, the activation of the PD-1/PD-L1 pathway is immunosuppressive.

#### Production and function of sPD-L1

Soluble PD-L1 (sPD-L1) is the most well-studied soluble immune checkpoint. It is produced through two different mechanisms – alternative splicing of PD-L1 mRNA [35, 36] or cleavage of the membrane-bound PD-L1 protein [37–40]. sPD-L1 has been found to be immunosuppressive, inhibiting T cell secretion of interferon gamma (IFN-γ) [35, 36] and interleukin 2 (IL-2) [36] and inducing the apoptosis of CD4^+^ [41, 42] and CD8^+^ [38, 42] T cells. Like its membrane counterpart, sPD-L1 can bind to PD-1 [36, 40, 43]. sPD-L1 can also bind to anti-PD-L1 monoclonal antibodies, thus potentially inducing resistance to anti-PD-L1 therapy [43].

#### sPD-L1 expression in *cancer* patient plasma/serum and association with tumor stage

Most studies have found sPD-L1 to be elevated in cancer patients compared to healthy donors, with higher levels observed in patients with non-small cell lung cancer (NSCLC) [44–47], small cell lung cancer [48], gastric cancer [49, 50], hepatocellular carcinoma [51–53], colorectal cancer [54], nasopharyngeal carcinoma [55], differentiated thyroid carcinoma [56], glioma [22], basal cell carcinoma [57], renal cell carcinoma [58], prostate cancer [59], and ovarian cancer [23, 60, 61]. However, some studies have reported no difference in the levels of sPD-L1 between healthy donors and patients with a variety of tumors including NSCLC [62], glioma [20], esophageal cancer [63], triple-negative breast cancer [64], and bladder cancer [21], and a few studies have described lower levels of sPD-L1 in patients with nasopharyngeal carcinoma [65], early breast cancer [66, 67], gastric carcinoma [68], hepatocellular carcinoma [69], and clear cell renal cell carcinoma [70] compared to healthy donors.

Among cancer patients, most studies (*n* = 17) have found higher sPD-L1 levels to be associated with a higher histological tumor grade and/or more advanced disease stage. sPD-L1 is associated with a higher histological tumor grade/stage of cancer in patients with gastric cancer [50], hepatocellular carcinoma [69, 71], glioma [20, 22], nasopharyngeal carcinoma [55], triple-negative breast cancer [64], renal cell carcinoma [72], and clear cell renal cell carcinoma [41]. Other studies have also reported a correlation between elevated sPD-L1 and more advanced disease. For example, higher sPD-L1 correlated with larger tumor size and greater venous invasion in patients with hepatocellular carcinoma [71], and with a larger tumor size and the presence of cervical lymph node metastasis in patients with differentiated thyroid carcinoma [56]. In patients with clear cell renal cell carcinoma, higher sPD-L1 was associated with larger tumors and increased tumor necrosis [41], and the presence of metastatic disease [70]. Higher sPD-L1 also correlated with the presence of metastasis in patients with renal cell carcinoma [72] and with the presence of liver metastasis in patients with NSCLC [73]. Elevated sPD-L1 also associated with the presence of muscle invasive disease and metastasis in patients with bladder cancer [21], lymph node metastasis in patients with colorectal cancer [54], higher Gleason scores in prostate cancer [59], less differentiated tumors and increased invasion and metastasis in renal cell carcinoma patients [58], and a greater residual tumor burden in patients with ovarian cancer [60]. Only a small number of reports (*n* = 4) have shown no association between sPD-L1 and tumor stage (in epithelial ovarian cancer [60], gastric cancer [74], hepatocellular carcinoma [51], and lung cancer [75]). Many variables exist among these studies, such as the number of patients evaluated, the material measured (serum or plasma), the assay used (ELISA or multiplex assay), and a high degree of variation in the cutoff values of sPD-L1 that were used to stratify patients into “high” vs “low” groups. Differences in any of these variables could explain why a few studies found conflicting results; however, these studies on sPD-L1 collectively suggest that levels are elevated in cancer patients compared to healthy donors, and that elevated levels are associated with a higher histological tumor grade and/or more advanced cancer stage. The prognostic value of sPD-L1 as an indicator of clinical response to ICI and conventional therapies will be addressed in subsequent sections.

#### Baseline sPD-L1 as an *indicator* of clinical response to ﻿ICI

**Fig. 2:**
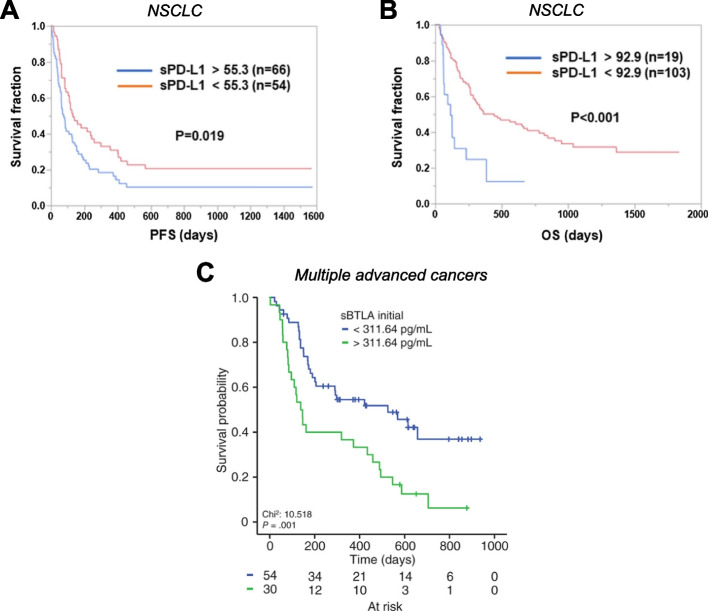
Elevated baseline levels of soluble immune checkpoints correlate with worse response to immune checkpoint therapy. Kaplan–Meier curves showing (**A**) progression-free survival (PFS) and (**B**) overall survival (OS) in NSCLC patients treated with anti-PD-1 or anti-PD-L1 monotherapy based on pre-treatment plasma sPD-L1 levels. Pre-treatment levels of sBTLA associated with OS of patients with advanced cancers treated with anti-PD-1 or the combination of anti-PD-1 plus anti-CTLA4 or other immune checkpoint inhibitors (**C**). Panels (**A**) and (**B**) modified from Himuro, Cancer Immunol Immunother, 2023 [78]. International Journal of Cancer published by John Wiley & Sons Ltd on behalf of UICC. Creative Commons CC-BY-NC license Copyright © 2023, Himuro et al., under exclusive licence to Springer-Verlag GmbH Germany, part of Springer Nature. Panel (**C**) modified from Gorgulho, Int J Cancer, 2021 [83]. © 2021 Gorgulho et al. International Journal of Cancer published by John Wiley & Sons Ltd on behalf of UICC. Creative Commons CC-BY-NC license

Eight studies involving 1,067 patients with solid tumors have reported that elevated levels of sPD-L1 at baseline are statistically associated with poor clinical response to ICI (Table 1). This is seen in patients treated with anti-PD-1 (e.g., nivolumab and pembrolizumab), anti-CTLA4 (e.g., ipilimumab), and anti-PD-L1 (e.g., durvalumab and atezolizumab) therapies, along with those treated with a combination of multiple ICI. Specifically, among NSCLC patients treated with anti-PD-1, higher baseline sPD-L1 levels correlated with a worse response rate and a shorter time to treatment failure (median: 1.48 vs 5.36 months) [76]. In this population, higher baseline sPD-L1 levels also correlated with shorter progression-free survival (PFS) [77] and overall survival (OS) [76]. A similar study in NSCLC patients treated with anti-PD-1 or anti-PD-L1 monotherapy found that elevated baseline sPD-L1 correlated with both shorter PFS (median: 76 vs 132 days, *p* = 0.019, Fig. 2A) and OS (median: 115 vs 444 days, *p* < 0.001, Fig. 2B) [78]. Elevated baseline sPD-L1 also associated with shorter PFS (median: 1.7 vs 2.1 months) and OS (median: 4.1 vs 8.9 months) in gastric cancer patients treated with anti-PD-1 [79], and with shorter OS (median: 24.6 vs > 40 months) and lower objective response rate (ORR) in metastatic renal cell carcinoma patients treated with nivolumab [80]. In another study of NSCLC patients treated with pembrolizumab or nivolumab, high baseline sPD-L1 associated with shorter PFS (median: 57 vs 177 days) and OS (median: 182 vs > 1000 days) [73]. In the same study, high baseline sPD-L1 also associated with a lower disease control rate, defined as the percent of patients with complete response (CR), partial response (PR), or stable disease (SD) (37% vs 57%) [73]. High baseline sPD-L1 also associated with shorter OS in urothelial cancer patients treated with atezolizumab or pembrolizumab [81]. Finally, Oh et al. showed that elevated baseline sPD-L1 correlated with shorter PFS (median: 2.9 vs 6.3 months) and OS (median: 7.4 vs 13.3 months) and a lower disease control rate (58% vs 79%) in patients with a variety of cancers who were treated with nivolumab, pembrolizumab, ipilimumab, durvalumab, atezolizumab or combination therapy [82].

**Table 1:** Baseline levels of sPD-L1 as an indicator of clinical response to immune checkpoint therapy

						**Association with Clinical Outcome**			
**Analyte**	**Cancer Type (n)**	**Treatment**	**Material**	**Method**	**Cutoff**	**Response**	**PFS**	**OS**	**Reference**
↑ sPD-L1	NSCLC (*n* = 39)	αPD-1	Plasma	ELISA, *PDCD1LG1* (Cloud-Clone Corp)	3.357 ng/ml	↓ (*p* = 0.0069)	↓ (*p* = 0.032)	↓ (*p* = 0.040)	[76]
	NSCLC (*n* = 51)	αPD-1	Plasma	ELISA, *SEA788Hu* (Cloud-Clone Corp)	0.156 ng/ml		↓ (*p* = 0.004)		[77]
	NSCLC (*n* = 122)	αPD-1 or αPD-L1	Plasma	ELISA (R&D)	92.9 pg/ml OS, 55.3 pg/ml PFS		↓ (*p* = 0.009)	↓ (*p* = 0.007)	[78]
	NSCLC (*n* = 233)	αPD-1	Serum	ELISA, *DB7H10* (R&D)	90 pg/ml	↓ *p* = 0.0158	↓ (*p* = 0.011)	↓ (*p* < 0.001)	[73]
	Gastric (*n* = 439)	αPD-1	Plasma	Automated immuno-assay system (HISCL, Sysmex)	295 pg/ml OS, 286 pg/ml PFS		↓ (*p* = 0.008)	↓ (*p* < 0.001)	[79]
	Metastatic renal cell (*n* = 43)	αPD-1	Serum	ELISA (HI-1000 system, Sysmex)	0.23 ng/ml	↓ (*p* = 0.0191)	*ns*	↓ (*p* = 0.0323)	[80]
	Urothelial (*n* = 12)	αPD-L1 or αPD-1	Serum	ELISA, *DB7H10* (R&D)	90 pg/ml			↓ (*p* = 0.040)	[81]
	Multiple cancers (*n* = 128)	αPD-1, αPD-L1, αCTLA4 or combination therapy	Serum	ELISA, *BMS2212* (Invitrogen)	11.0 pg/μl	↓ (*p* = 0.013)	↓ (*p* = 0.023)	↓ (*p* = 0.005)	[82]

*Abbreviations: PFS* Progression-free survival, *OS* Overall survival, *NSCLC* Non-small cell lung cancer, *ns* Not significant, *sPD-L1* Soluble programmed cell death-ligand 1

While most studies have shown that elevated baseline sPD-L1 is indicative of a worse response to ICI (Table 1), a few (*n* = 3) have found baseline sPD-L1 to have a negligible prognostic value, while two others have reported high levels to positively associate with patient outcome. Castello et al. found that baseline sPD-L1 levels did not impact PFS or OS in NSCLC patients treated with nivolumab, pembrolizumab or a combination of nivolumab and ipilimumab [84], while Ando et al. observed that baseline sPD-L1 was not correlated with OS in patients with NSCLC, gastric cancer, or bladder cancer who received nivolumab or pembrolizumab [85]. Among NSCLC patients treated with nivolumab, there were no differences in baseline sPD-L1 levels between responders and non-responders [86]. Incorvaia et al. showed that high baseline sPD-L1 correlated with longer PFS (median: 19 vs 9 months) in clear cell renal cell carcinoma patients treated with nivolumab [87], while Zhao et al. observed that high baseline sPD-L1 correlated with a better response to anti-PD-1 or anti-PD-L1 monotherapy in patients with a variety of cancers [88]. As detailed above, numerous investigators have evaluated the association between baseline levels of sPD-L1 and association with patient outcome following ICI. While there are some conflicting findings, which may be impacted by heterogeneity of the patient populations evaluated, the type of material and assays utilized to measure sPD-L1, and variation in the cut points used in which to stratify patients into groups, the vast majority of these studies indicate that lower levels of sPD-L1, prior to initiating ICI, can identify patients with a variety of cancers with improved clinical responses following ICI.

#### Post-treatment levels of sPD-L1 after ICI as an *indicator* of clinical response

**Fig. 3:**
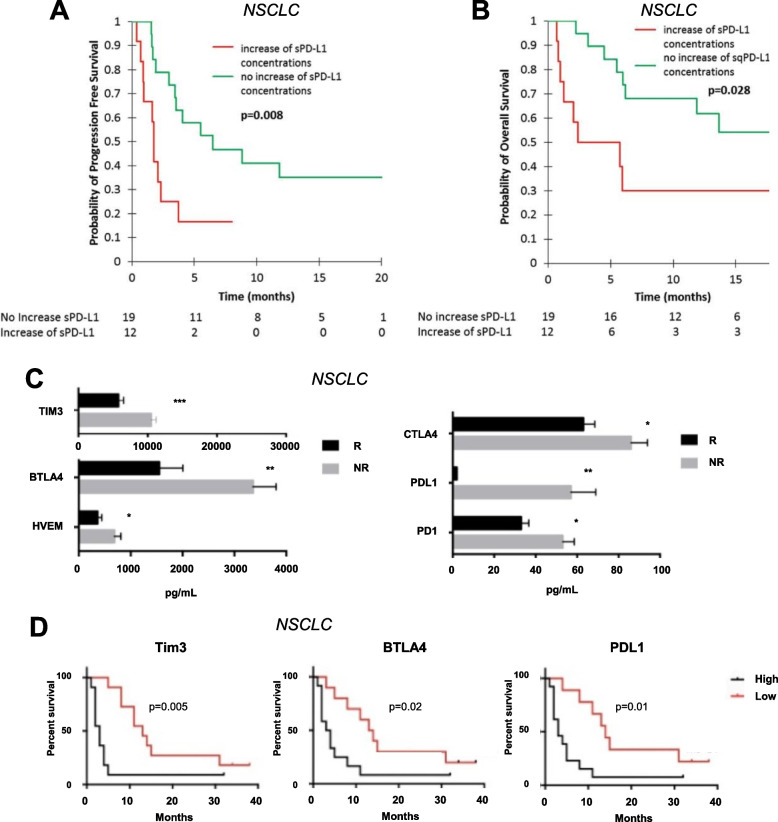
Post-treatment levels of soluble immune checkpoints after immune checkpoint therapy associate with patient response. The change in circulating levels of sPD-L1 at the first tumor evaluation (2 months after the initiation of nivolumab treatment) compared to baseline associated with progression-free survival (PFS) (**A**) and overall survival (OS) (**B**) in advanced NSCLC patients. Post treatment levels of the soluble immune checkpoints sTIM3, sBTLA4, sHVEM, sCTLA4, sPD-L1 and sPD-1 were higher after treatment with nivolumab in non-responding (NR) vs responding (R) patients with NSCLC (**C**). In this study, post treatment levels of sPD-L1, sTIM3, and sBTLA4 also associated with PFS (**D**). Panels (**A** and **B**) from Constantini, Oncoimmunology April 20, 2018 [86]. Reprinted by permission of the publisher Taylor & Francis Ltd., http://www.tandfonline.com. Panels (**C** and **D**) modified from Zizzari, J Pers Med 2020 [90]*.* Copyright © 2020 by Zizzari et al. Licensee MDPI, Basel, Switzerland. This article is an open access article distributed under the terms and conditions of the Creative Commons Attribution (CC BY) license (http://creativecommons.org/licenses/by/4.0/)

Plasma and serum levels of soluble immune checkpoints can change upon treatment with ICI, and in some cases, the magnitude of this change associates with clinical response. Three studies reported that sPD-L1 increased upon ICI therapy [81, 84, 89], while two observed sPD-L1 to remain constant upon ICI treatment (Table S1) [77, 90]. In terms of clinical outcome, either a decrease or less of an increase in sPD-L1 upon treatment with ICI is correlated with improved clinical responses, with four studies (*n* = 204 patients) reporting similar findings (Table 2). In patients with NSCLC, gastric cancer, or bladder cancer treated with nivolumab or pembrolizumab, a greater decrease in sPD-L1 after four cycles of treatment correlated with a greater decrease in tumor size [85]. Similarly, in NSCLC patients treated with nivolumab, an increase in sPD-L1 after 2 months of treatment correlated with a lower ORR (17% in patients with increases vs 68% in patients with decreases or stable levels) and shorter PFS (median: 1.8 vs 6.5 months, Fig. 3A), and OS (median: 5.4 months vs not reached as of 20 months, Fig. 3B) [86]. In that study, high levels of sPD-L1 after 2 months of treatment also associated with poor response, with non-responders having higher levels of sPD-L1 than responders (median: 67.64 vs 32.94 pg/mL) [86]. In another study, patients with NSCLC who responded to nivolumab treatment had lower levels of sPD-L1 3 months after treatment compared to non-responders (Fig. 3C), and low sPD-L1 was correlated with longer PFS (Fig. 3D) [90]. NSCLC patients treated with pembrolizumab or nivolumab who had high levels of sPD-L1 after 6 weeks of treatment exhibited a shorter PFS (median: 64 vs 239 days) than patients with low levels of sPD-L1 at this timepoint [78]. In the same study, patients with high levels of sPD-L1 after 6 weeks of treatment exhibited shorter OS (median: 118 vs 653 days) than patients with levels below this threshold [78]. These studies collectively show that a reduction or stabilization in sPD-L1 levels after ICI associate with improved clinical outcomes.

**Table 2:** Post-treatment levels of sPD-L1 after immune checkpoint therapy as an indicator of clinical response

					**Direction Post-** **treatment**	**Association with Clinical Outcome**			
**Analyte**	**Cancer Type (n)**	**Treatment**	**Material**	**Method**		**Response**	**PFS**	**OS**	**Reference**
sPD-L1	NSCLC (*n* = 39)	αPD-1	Plasma	ELISA, *ab214565* (Abcam)	↓	↑ (*p* = 0.005)	↑ (*p* = 0.008)	↑ (*p* = 0.028)	[86]
	NSCLC (*n* = 22)	αPD-1	Serum	hIO Checkpoint 14-Plex ProcartaPlex Panel (Thermo Fisher Scientific)	↓	↑ (*p* < 0.01)	↑ (*p* = 0.01)		[90]
	NSCLC (*n* = 122)	αPD-1 or αPD-L1	Plasma	ELISA (R&D)	↓		↑ (*p* = 0.008)	↑ (*p* < 0.001)	[78]
	Multiple cancers (*n* = 21)	αPD-1	Plasma	DuoSet ELISA (R&D)	↓	↑ (*p *< 0.05)			[85]

*Abbreviations: PFS* Progression-free survival, *OS* Overall survival, *hIO* Human Immuno-Oncology, *NSCLC* Non-small cell lung cancer, *sPD-L1* Soluble programmed cell death-ligand 1

### sPD-1

#### Structure and function of membrane-bound PD-1

Programmed cell death protein 1 (PD-1) is expressed on T cells, B cells, and myeloid cells [34, 91–93]. It is a transmembrane glycoprotein that consists of an IgV-type extracellular domain, a stalk, a transmembrane domain, and a cytoplasmic domain [94–96]. The cytoplasmic domain contains two tyrosines, one of which forms an immunoreceptor tyrosine-based inhibitory motif (ITIM) while the other forms an immunoreceptor tyrosine-based switch motif (ITSM) [29–31]. The binding of PD-L1 to PD-1 induces the phosphorylation of the cytoplasmic ITSM on PD-1; a phosphorylated ITSM recruits SHP-1/2, which downregulates downstream pathways such as the PI3K-AKT-mTOR and RAS-MEK-ERK pathways [29–32]. The downregulation of these pathways suppresses T cell growth, survival, and proliferation [32–34]; thus, the activation of the PD-1/PD-L1 pathway is immunosuppressive.

#### Production and function of sPD-1

Soluble PD-1 (sPD-1) is produced through alternative splicing of PD-1 mRNA [97] and is not nearly as well-studied as sPD-L1. Functional studies of sPD-1 have been performed both in vitro and in mice, with most of these studies, in contrast to membrane-bound PD-1, reporting sPD-1 to exhibit pro-inflammatory and anti-tumor effects; sPD-1 activated T lymphocytes [98–101], upregulated the expression of IFN-γ and tumor necrosis factor alpha (TNF-α) [98], and reduced expression of IL-10 [98]. These changes correlated with increased tumor cell lysis [100] and reduced tumor growth in several murine cancer models [98–100, 102]. However, one study reported that sPD-1 inhibited CD4^+^ T cell activation in the presence of dendritic cells [103]. In addition, sPD-1 can bind to PD-L1 and PD-L2 [102], but whether this interaction is immunosuppressive or immune-activating requires further study.

#### sPD-1 expression in *cancer* patient plasma/serum and association with tumor stage

Most studies have found sPD-1 to be elevated in cancer patients compared to healthy donors, with elevated levels reported in patients with NSCLC [47, 104], esophageal cancer [63], differentiated thyroid carcinoma [56], hepatocellular carcinoma [105], nasopharyngeal carcinoma [55], basal cell carcinoma [57], triple-negative breast cancer [64], prostate cancer [59], and ovarian cancer [23]. One study reported no difference between the levels of sPD-1 in healthy donors and early breast cancer patients [67], while a few others reported lower levels of sPD-1 in patients with nasopharyngeal carcinoma [65], gastric cancer [106], and gastric carcinoma [68].

Among cancer patients, higher sPD-1 levels in most reports (*n* = 5) are associated with more advanced cancers. Higher levels of sPD-1 associate with a higher histological tumor grade/stage of cancer in patients with triple-negative breast cancer [64], ovarian cancer [23], and gastric cancer [106]. Similarly, higher sPD-1 correlated with increased tumor invasion in patients with renal cell carcinoma [58] and with a larger tumor size and the presence of cervical lymph node metastasis in patients with differentiated thyroid carcinoma [56]; however, sPD-1 did not correlate with Gleason scores in patients with prostate cancer [59]. Only one study in patients with nasopharyngeal carcinoma found that lower levels of sPD-1 associated with more advanced stages of disease [65]. Thus, while there are some conflicting reports, the majority of studies on sPD-1 suggest that levels are elevated in cancer patients compared to healthy donors, and that higher levels are most typically associated with a more advanced cancer phenotype.

#### Baseline sPD-1 as an *indicator* of clinical response to ﻿ICI

Elevated levels of sPD-1 prior to initiation of ICI have been shown in two studies (with a total of 490 patients) to statistically correlate with a poor clinical response to therapy (Table S2). High baseline sPD-1 was correlated with shorter PFS in NSCLC patients treated with nivolumab [77] and with shorter OS (median: 5.7 vs 8.5 months) in gastric cancer patients treated with nivolumab [79]. In contrast, a single study found that high baseline sPD-1 correlated with longer PFS (median: 20.7 vs 6.9 months) and overall response in clear cell renal cell carcinoma patients treated with nivolumab [87]. Additional studies are needed to elucidate the role of baseline levels of sPD-L1 as an indicator of patient response to ICI.

#### Post-treatment levels of sPD-1 after ICI as an *indicator* of clinical response

Studies have reported conflicting findings regarding changes in sPD-1 upon ICI therapy, with some studies reporting an increase [107], decrease [90] or no change upon treatment (Table S1) [77]. Three studies, however, have shown that either a decline, or less of an increase in sPD-1 upon treatment with ICI associates with improved response to therapy (Table S3). Clear cell renal cell carcinoma patients responding to nivolumab experienced a decrease in sPD-1 (from a median of 13.25 to 1.23 ng/mL) after two cycles of treatment [87]; however, in that study, changes in sPD-1 after nivolumab treatment were not investigated in non-responders. In a different study in patients with NSCLC, responders experienced a decrease in sPD-1 after 3 months of nivolumab treatment, while sPD-1 levels remained constant in non-responding patients [90]. The authors also showed that responders had lower absolute levels of sPD-1 3 months after treatment than non-responders (*p* < 0.05, Fig. 3C) [90]. Among patients with NSCLC, gastric cancer, or bladder cancer treated with nivolumab or pembrolizumab, sPD-1 levels were typically higher after the second cycle of treatment than at baseline, and a greater rate of increase between the second and fourth cycle of treatment correlated with an increase in tumor size [107]. While these studies collectively suggest that a decrease, or lower levels of sPD-1 after ICI associate with improved clinical outcomes, two other studies have reported conflicting findings (Table S3). Tiako Meyo et al. found that increased (> 30%) or stable levels of sPD-1 after two cycles of nivolumab in NSCLC patients correlated with both longer PFS (median: 121 vs 50 days) and OS (median: 450 vs 153 days) [77]. In addition, Himuro et al. showed that NSCLC patients treated with pembrolizumab or nivolumab who had high levels of sPD-1 after 6 weeks of treatment exhibited a longer OS (median: 821 vs 183 days) than patients with levels below this threshold [78]. These studies demonstrate that changes or post-treatment levels in sPD-1 do not consistently associate with patient outcomes following ICI and suggest that further evaluations of this soluble checkpoint are needed.

### sCTLA4

#### Structure and function of membrane-bound CTLA4

Cytotoxic T-lymphocyte-associated antigen 4 (CTLA4) is expressed on T cells [108–110]. It is a transmembrane glycoprotein that consists of an IgV-type extracellular domain, a transmembrane domain, and a cytoplasmic domain [111–113]. The binding of CTLA4 to CD80 (or to CD86) suppresses T cell growth, survival, and proliferation [109, 110, 114]. However, the intracellular signaling mechanisms of the CTLA4/CD80-CD86 pathway remain unclear. The cytoplasmic domain of CTLA4 has been shown to interact with protein phosphatase 2A (PP2A), SHP-2, and PI3K, interactions which may affect downstream pathways such as the PI3K-AKT-mTOR and RAS-MEK-ERK pathways [31, 115–119].

#### Production and function of sCTLA4

Soluble CTLA4 (sCTLA4) is produced through alternative splicing of CTLA4 mRNA [120, 121]. Like its membrane counterpart, sCTLA4 has been found to have immunosuppressive functions, with the addition of recombinant sCTLA4 to human peripheral blood mononuclear cells (PBMCs) in vitro reducing CD8^+^ and CD4^+^ T cell proliferation and inhibiting the secretion of IFN-γ, IL-17A, and IL-10 [122]. In this study, antibody blockade of sCTLA4 increased effector cytokine secretion to partially reverse this immunosuppression [122]. Another group similarly showed that antibody blockade of sCTLA4 induced T cell proliferation and cytokine (IFN-γ and IL-17) secretion by human PBMCs in vitro [123]. sCTLA4 can bind to CD80 and CD86, and this interaction was found to inhibit a mixed lymphocyte reaction [121]. This report also suggested that binding of sCTLA4 to CD80 and CD86 could compete with and inhibit the binding of CD80 and CD86 to the co-stimulatory CD28 molecule [121].

#### sCTLA4 expression in *cancer* patient plasma/serum and association with tumor stage

Several studies have found sCTLA4 to be elevated in cancer patients compared to healthy donors, with higher levels reported in patients with NSCLC [104], nasopharyngeal carcinoma [55], basal cell carcinoma [57], breast cancer [124], and ovarian cancer [23]. Only a few studies have reported lower levels of sCTLA4 in cancer patients (early breast cancer [66, 67], nasopharyngeal carcinoma [65], and clear cell renal cell carcinoma [125]) than in healthy donors. A single study, in patients with hepatocellular carcinoma with chronic hepatitis C infection, found higher concentrations of sCTLA4 to correlate with a later TNM stage and a larger tumor size [126]. Overall, most studies suggest that sCTLA4 is elevated in cancer patients compared to healthy donors; however, further work is needed to understand its association with cancer stage.

#### Baseline and post-treatment levels of sCTLA4 as an *indicator* of clinical response to ICI

Only one study has reported on the association between levels of sCTLA4 prior to ICI in solid tumors other than melanoma and clinical response (Table S2). Increased levels of sCTLA4 prior to treatment correlated with shorter OS (median: 5.3 vs 7.9 months) in gastric cancer patients treated with nivolumab [79]. Zizzari et al. showed that the levels of sCTLA4 were not significantly changed in NSCLC patients upon treatment with nivolumab (Table S1) [90]. The same study observed that patients with NSCLC who responded to nivolumab had lower levels of sCTLA4 3 months after treatment than non-responders (Table S3, Fig. 3C) [90].

### sCD80

#### Structure and function of membrane-bound CD80

CD80 is expressed on dendritic cells, macrophages, B cells, and tumor cells [127–130]. It is a transmembrane glycoprotein that consists of IgC and IgV-type extracellular domains, a transmembrane domain, and a cytoplasmic domain [129, 130]. The binding of CTLA4 to CD80 (or to CD86) suppresses T cell growth, survival, and proliferation [109, 110, 114]. However, the intracellular signaling mechanisms of the CTLA4/CD80-CD86 pathway remain unclear. The cytoplasmic domain of CTLA4 has been shown to interact with PP2A, SHP-2, and PI3K, interactions which may affect downstream pathways such as the PI3K-AKT-mTOR and RAS-MEK-ERK pathways [31, 115–119]. In addition to CTLA4, CD80 (and CD86) can bind to CD28; this is an immunostimulatory interaction that induces T cell proliferation [110, 131].

#### Production and function of sCD80

Soluble CD80 (sCD80) is produced through alternative splicing of CD80 mRNA [132, 133], and studies regarding its function are conflicting. Kakoulidou et al. showed that recombinant sCD80 inhibited both a mixed lymphocyte reaction and anti-CD3-induced T cell proliferation of human PBMCs [132]. However, multiple other studies, both in vitro and in vivo*,* have found sCD80 to have anti-tumor effects. In vitro, Haile et al. demonstrated that sCD80 bound to PD-L1, inhibiting the PD-L1-PD-1 immunosuppressive pathway and restoring T cell secretion of IFN-γ [134]. Concurrently, sCD80 induced the co-stimulation of CD28 [134, 135]. In vivo, sCD80 reduced tumor growth and induced the recruitment of tumor-infiltrating lymphocytes (TILs) in a murine model of colon carcinoma [136]. Similarly, Sturmhoefel et al. showed that sCD80 reduced tumor growth and increased OS in mice both when given as an independent treatment and as a vaccine adjuvant [137]. That study found the anti-tumor response of sCD80 to be dependent on CD8^+^ T cells but independent of CD4^+^ T cells and IFN-γ [137].

#### sCD80 expression in *cancer* patient plasma/serum and association with tumor stage

Studies reporting on differences in the level of sCD80 between cancer patients and healthy donors are conflicting, with higher levels observed in patients with NSCLC [104] and nasopharyngeal carcinoma [55], but lower levels in a different study of patients with nasopharyngeal carcinoma [65], and in patients with early breast cancer [66]. In addition, similar levels have been reported between healthy donors and patients with soft tissue sarcoma and benign tumors [138], or early breast cancer [67]. To date, the association between sCD80 levels and tumor stage has been reported in only two studies; higher sCD80 levels correlated with increased tumor invasion in patients with NSCLC [45] and with increased invasion and less tumor differentiation in patients with renal cell carcinoma [58]. Thus, while sCD80 levels do not consistently differ between healthy donors and cancer patients, higher levels within cancer patients appear to associate with a more advanced cancer stage.

#### Baseline and post-treatment levels of sCD80 after ICI as an *indicator* of clinical response

No studies to date have evaluated the association between baseline levels or changes in levels of sCD80 after ICI and clinical response. Only one study has reported on changes in sCD80 levels after ICI, with Zizzari et al. showing that the levels were not significantly changed in NSCLC patients upon treatment with nivolumab (Table S1) [90].

### sTIM3

#### Structure and function of membrane-bound TIM3

T cell immunoglobulin and mucin domain-containing protein 3 (TIM3) is expressed on T cells [139]. It is a transmembrane glycoprotein that consists of IgV-type and mucin-like extracellular domains, a transmembrane domain, and a cytoplasmic domain [139]. TIM3 has several binding partners, including GAL9 [140, 141]. The binding of TIM3 to GAL9 induces the phosphorylation of at least one of the cytoplasmic tyrosines on TIM3 [142]. Further intracellular signaling is unclear, but the TIM3/GAL9 pathway induces T cell death and MDSC expansion [140, 143, 144].

#### Production and function of sTIM3

Soluble TIM3 (sTIM3) is produced through both alternative splicing of TIM3 mRNA [145, 146] and cleavage of the membrane-bound TIM3 protein [147]. Few studies have investigated the function of sTIM3, and findings are conflicting. Sabatos et al. reported that sTIM3-Ig bound to the same ligands as membrane-bound TIM3, and that mice treated with a fusion protein of sTIM3 (sTim-3-Ig) had hyperproliferation of T_h_1 cells and increased release of T_h_1 cytokines IL-2 and IFN-γ [145]. The authors hypothesized that the normal interaction between TIM3 and TIM3 ligands is immunosuppressive, and the binding of sTIM-3-Ig to TIM3 ligands can block this inhibitory response, thus promoting anti-tumor effects [145]. In contrast, Geng et al. found sTIM3 to have immunosuppressive functions in both in vitro and in vivo studies [146]. In vitro, sTIM3 inhibited T cell proliferation in response to antigen-specific stimulation and anti-CD3/anti-CD28 costimulation*,* and inhibited the production of IL-2 and IFN-γ. Similarly, in murine models, sTIM3 reduced anti-tumor cytotoxic T lymphocyte activity, the number of TILs, and the expression of T_h_1 cytokines IL-2, IFN-γ, and TNF-β [146]. In these studies, sTIM3 also increased tumor growth in a murine model of hepatocarcinoma [146].

#### sTIM3 expression in *cancer* patient plasma/serum and association with tumor stage

Most studies that have evaluated sTIM3 levels have found them to be higher in cancer patients compared to healthy donors, with increased levels observed specifically in patients with NSCLC [104], hepatocellular carcinoma [148], oral squamous cell carcinoma [149], differentiated thyroid carcinoma [56], basal cell carcinoma [57], gastric cancer [150], and osteosarcoma [151]. Only two studies have reported lower levels of sTIM3, both in patients with early breast cancer, compared to those of healthy donors [66, 67], while two others found no difference in the levels between healthy donors and patients with nasopharyngeal carcinoma [55, 65].

Among cancer patients, higher sTIM3 has consistently been reported (in seven different studies) to associate with a higher histological tumor grade/stage of cancer. This is true in NSCLC [104], hepatocellular carcinoma [152], oral squamous cell carcinoma [149], gastric cancer [150], clear cell renal cell carcinoma [153], and osteosarcoma [151]. Higher sTIM3 also correlated with a larger tumor size and the presence of distant metastases in patients with osteosarcoma [151], and with larger tumor size, later TNM stage, and the presence of cervical lymph node metastases in patients with differentiated thyroid carcinoma [56]. Overall, while sTIM3 may not consistently differ in level between healthy donors and cancer patients, higher levels within patients with cancer consistently associate with more advanced disease.

#### Baseline and post-treatment levels of sTIM3 after ICI as an *indicator* of clinical response

One study found that elevated baseline sTIM3 correlated with a better response to anti-PD-1 or anti-PD-L1 monotherapy in patients with a variety of cancers (Table S2) [88]. Zizzari et al. showed that the levels of sTIM3 were not significantly changed in NSCLC patients upon treatment with nivolumab (Table S1) [90]; however, the same study observed that patients with NSCLC who responded to nivolumab had lower levels of sTIM3 3 months after treatment than non-responders (Table S3, Fig. 3C), and low concentrations of sTIM3 were correlated with longer PFS (Fig. 3D) [90]. Additional cohorts of patients are needed to confirm the relevance of pre- and post-treatment levels of sTIM3 as an indicator of response to ICI.

### sLAG3

#### Structure and function of membrane-bound LAG3

Lymphocyte activation gene 3 (LAG3) is expressed on T cells and NK cells [154, 155]. It is a transmembrane glycoprotein that consists of four immunoglobulin superfamily extracellular domains, a transmembrane domain, and a cytoplasmic domain [154]. The cytoplasmic domain contains a “KIEELE” motif, the lysine of which (K468) is thought to be important for intracellular signaling [156]. Though the exact intracellular signaling mechanisms remain unclear, the binding of membrane-bound LAG3 to MHC class II molecules induces the suppression of T cell function and proliferation [155–160].

#### Production and function of sLAG3

Soluble LAG3 (sLAG3) is produced through cleavage of the membrane-bound LAG3 protein [161, 162], and several studies, in contrast to membrane-bound LAG3, have shown sLAG3 to have immunostimulatory functions [163–172]. sLAG3 binds to MHC class II molecules [168, 169, 172] and induces dendritic cell maturation [166, 168–170, 172], increases production of IL-12, TNF-α, and IFN-γ [163, 166–169], and induces T cell proliferation [163, 164, 167, 168]. In murine models, sLAG3 reduced tumor growth [163, 165, 171] and increased the duration of OS [163]. One study also showed that high levels of sLAG3 in gastric cancer patients correlated with increased immune activation, as evidenced by higher levels of IL-12 and IFN-γ [163]. Other studies have reported that the cleavage of membrane LAG3 is required for T cell proliferation [161], and the induction of an anti-tumor response upon anti-PD-1 treatment [173]. However, one study suggested that sLAG3 itself does not cause this immunostimulatory effect but is instead an inert byproduct of LAG3 cleavage [161].

#### sLAG3 expression in *cancer* patient plasma/serum and association with tumor stage

Most studies evaluating levels of sLAG3 have found this checkpoint to be elevated in cancer patients compared to healthy donors, with higher levels described in patients with NSCLC [104], hepatocellular carcinoma [51], nasopharyngeal carcinoma [55, 65], differentiated thyroid carcinoma [56], pancreatic ductal adenocarcinoma [174], and basal cell carcinoma [57]. To date, only a single study reported no difference in sLAG3 levels between healthy donors and patients with early breast cancer [67], while one other study reported lower levels in patients with gastric cancer compared to healthy donors [163]. Among cancer patients, sLAG3 has been reported in three studies to associate with more advanced disease, with higher levels associating with an advanced cancer stage in hepatocellular carcinoma [51] and clear cell renal cell carcinoma [153], and the presence of cervical lymph node metastasis in patients with differentiated thyroid carcinoma [56]. Only one report has found that lower sLAG3 levels were associated with a more advanced stage in patients with NSCLC [175]. Overall, higher levels of sLAG3 are observed in cancer patients than healthy individuals, and higher levels associate with a more advanced cancer phenotype; however, further studies are warranted.

#### Baseline and post-treatment levels of sLAG3 after ICI as an *indicator* of clinical ﻿response

A single study reported that elevated baseline sLAG3 was correlated with shorter PFS and OS in head and neck squamous cell carcinoma patients treated with chemotherapy or nivolumab (Table S2) [176]. Another study, by Zizzari et al., showed that the levels of sLAG3 increased in NSCLC patients upon treatment with nivolumab (Table S1) [90]. The same study also reported that responders experienced no change in sLAG3 while non-responders experienced an increase in this soluble immune checkpoint after six cycles of nivolumab treatment (Table S3) [90]. Additional studies are needed to elucidate the role of sLAG3 as an indicator of clinical response to ICI.

### sB7-H3

#### Structure and function of membrane-bound B7-H3

B7-H3 is expressed on APCs (such as dendritic cells and monocytes), T cells, B cells, NK cells, and tumor cells [177, 178]. It is a transmembrane glycoprotein that consists of IgC and IgV-type extracellular domains, a transmembrane domain, and a cytoplasmic domain [177]. A second isoform, called 4Ig-B7-H3, contains two pairs of IgC-IgV extracellular domains as opposed to simply one pair [178–180]. Both isoforms of B7-H3 (2Ig-B7-H3 and 4Ig-B7-H3) are present in humans, but 4-Ig-B7-H3 is predominant [179, 180]. Little is known about B7-H3 intracellular signaling – the binding partner of B7-H3 is unknown, and B7-H3 signaling has been shown to have both immunosuppressive and immunostimulatory effects [177, 178, 180–184].

#### Production and function of sB7-H3

Soluble B7-H3 (sB7-H3) is produced through both alternative splicing of B7-H3 mRNA [185] and cleavage of the membrane-bound B7-H3 protein [186, 187]. Only two studies have evaluated the function of sB7-H3, with both reporting immunosuppressive functions. One group found that sB7-H3 inhibited T cell proliferation and cytokine production (IL-2 and IFN-γ) in vitro [185], while another found that sB7-H3 increased TLR4 expression, which in turn activated NF-kB signaling and then induced IL-8 and VEGF expression [188]. As a result, sB7-H3 induced the migration and invasion of pancreatic cancer cells in vitro and led to increased lung metastasis in a murine model of pancreatic cancer [188].

#### sB7-H3 expression in *cancer* patient plasma/serum and association with tumor stage

Seven different studies have reported sB7-H3 to be elevated in cancer patients compared to healthy donors; elevated levels have been observed in patients with NSCLC [189], colorectal carcinoma [187], gastric adenocarcinoma [190], hepatocellular carcinoma [185, 191], non-muscle-invasive bladder cancer [192], and osteosarcoma [193]. Only a single report found lower levels of sB7-H3 in patients with clear cell renal cell carcinoma compared to those of healthy donors [125], while another observed no difference in levels between healthy donors and cancer patients with glioma [20].

Among cancer patients, multiple studies (*n* = 6) have consistently shown that higher sB7-H3 levels associated with a higher histological tumor grade/stage of cancer. This has been demonstrated in patients with NSCLC [189], gastric adenocarcinoma [190], hepatocellular carcinoma [191], glioma [20], ovarian cancer [194], and osteosarcoma [193]. Higher sB7-H3 also correlated with larger tumor size, nodal metastasis, and the presence of distant metastasis in patients with NSCLC [189], greater metastasis and less tumor differentiation in patients with osteosarcoma [193], and with larger tumor size, greater vascular invasion, and less tumor differentiation in patients with hepatocellular carcinoma [191]. Overall, higher levels of sB7-H3 are observed in patients with cancer compared to healthy controls, and higher levels of sB7-H3 associate with more advanced disease. No studies to date have evaluated the association between baseline levels or changes in levels of sB7-H3 after ICI as an indicator of clinical response.

### sBTLA

#### Structure and function of membrane-bound BTLA

B and T lymphocyte attenuator (BTLA) is expressed on T cells, B cells, and APCs (including dendritic cells and macrophages) [195, 196]. It is a transmembrane glycoprotein that consists of an IgV-type extracellular domain, a transmembrane domain, and a cytoplasmic domain [195]. The cytoplasmic domain contains three tyrosines, two of which form ITIMs [195]. BTLA intracellular signaling is very similar to that of PD-1. The binding of BTLA to HVEM induces the phosphorylation of both cytoplasmic ITIMs on BTLA; as a result, BTLA recruits SHP-1/2 [197–199]. The targets of SHP-1/2 after recruitment to BTLA are unknown [200], but the BTLA/HVEM signaling pathway induces the suppression of T cell activation and proliferation [195, 196, 198, 199, 201].

#### Production and function of sBTLA

Soluble BTLA (sBTLA) is produced through alternative splicing of BTLA mRNA [202]. One study evaluated the function of sBTLA and, in contrast to membrane-bound BTLA, reported it to have pro-inflammatory and anti-tumor effects. Han et al. demonstrated that sBTLA can bind to HVEM, and reduced IL-10 and TGF-β expression in a murine model of cervical cancer, but did not sufficiently eliminate the tumor [203]. The authors identified that the combination of sBTLA and an HSP70 vaccine significantly improved the anti-tumor immune response, with combination treatment increasing expression of IL-2, IFN-γ, and CD8^+^ TILs, and reducing expression of IL-10, TGF-β, and Foxp3 [203]. This study demonstrates that by binding to HVEM, sBTLA can inhibit the immunosuppressive BTLA-HVEM interaction and in turn exert anti-tumor effects.

#### sBTLA expression in *cancer* patient plasma/serum and association with tumor stage

Five studies have evaluated the expression of sBTLA in cancer patients, with most reporting elevated levels compared to healthy donors. sBTLA was elevated in patients with nasopharyngeal carcinoma [55] and pancreatic ductal adenocarcinoma [174], as well as in patients with a variety of other cancers (including NSCLC, urogenital tract cancer, gastrointestinal cancer, and head and neck cancer) compared to healthy donors [83]. To date, a single study reported no difference in sBTLA between patients with early breast cancer and healthy controls [67], while another described lower levels in patients with nasopharyngeal carcinoma than healthy donors [65]. Notably, no studies have reported on the association between circulating levels of sBTLA and cancer stage.

#### Baseline and post-treatment levels of sBTLA after ICI as an *indicator* of clinical response

Gorgulho et al. showed that elevated levels of sBTLA at baseline correlated with shorter OS (median: 138 vs 526 days) in patients with a variety of cancers (*n* = 84) treated with nivolumab, pembrolizumab, combination treatment (nivolumab and ipilimumab) or other ICI (Table S2, Fig. 2C) [83]. This same study also showed that levels of sBTLA remained constant in patients with a variety of cancers who were treated with anti-PD-1, combination therapy (of anti-PD-1 plus anti-CTLA4) or other ICI (Table S1) [83]. Zizzari et al. similarly reported that the levels of sBTLA were not significantly changed in NSCLC patients upon treatment with nivolumab (Table S1) [90]. Zizzari et al. also observed that patients with NSCLC who responded to nivolumab had lower levels of sBTLA 3 months after treatment than non-responders (Table S3, Fig. 3C), and low concentrations of sBTLA were correlated with longer PFS (Fig. 3D) [90]. Similarly, Gorgulho et al. demonstrated that low levels of sBTLA post therapy associated with improved OS at both an early (*p* = 0.018) and late (*p* = 0.009) time point in patients with a variety of cancers who were treated with anti-PD-1, combination therapy (of anti-PD-1 plus anti-CTLA4) or other ICI (Table S3) [83]. Thus, low levels of sBTLA, both before and after ICI, are associated with improved clinical response. Further studies in additional patient populations treated with ICI are needed to confirm these findings.

### sHVEM

#### Structure and function of membrane-bound HVEM

Herpesvirus entry mediator (HVEM) is expressed on T cells, B cells, NK cells, monocytes, dendritic cells, and tumor cells [204–206]. It is a transmembrane glycoprotein that consists of an extracellular domain with four cysteine-rich domains, a transmembrane domain, and a cytoplasmic domain [204, 207]. The binding of BTLA to HVEM induces the phosphorylation of both cytoplasmic ITIMs on BTLA; as a result, BTLA recruits SHP-1/2 [197–199]. The targets of SHP-1/2 after recruitment to BTLA are unknown [200], but the BTLA/HVEM signaling pathway induces the suppression of T cell activation and proliferation [195, 196, 198, 199, 201]. In addition to BTLA, HVEM can also bind to other molecules, such as LIGHT; these interactions can have immunostimulatory effects [208–210].

#### Production and function of sHVEM

There are few studies reporting on the production and function of soluble HVEM (sHVEM). sHVEM is thought to be produced through cleavage of the membrane-bound HVEM protein [211]; however, the exact role of sHVEM is unclear. HVEM has multiple binding partners, including BTLA and LIGHT, with the HVEM-BTLA interaction serving as immunosuppressive, and the HVEM-LIGHT interaction immune-stimulatory [199, 212, 213]. sHVEM binds with higher affinity to LIGHT than BTLA [213, 214]; thus, two groups have hypothesized that sHVEM, by binding to LIGHT and interfering with the HVEM-LIGHT immune-stimulatory pathways, primarily has immunosuppressive effects [215, 216].

#### sHVEM expression in *cancer* patient plasma/serum and association with tumor stage

Six studies have evaluated sHVEM level in cancer patients as compared to healthy donors, with four reporting higher levels in cancer patients (with gastric cancer [211, 216], hepatocellular carcinoma [215], and nasopharyngeal carcinoma [55]), and two reporting lower levels in cancer patients (with nasopharyngeal carcinoma [65] and early breast cancer [67]). The association between sHVEM and cancer stage has only been evaluated in a single study; here, hepatocellular carcinoma patients with higher sHVEM levels had a more advanced stage of cancer [215]. Overall, most studies suggest that sHVEM is elevated in cancer patients compared to healthy donors; however, further work is needed to understand its association with tumor stage.

#### Baseline and post-treatment levels of sHVEM after ICI as an *indicator* of clinical response

No studies have evaluated the association between baseline levels of sHVEM and clinical response to ICI. One study, by Zizzari et al., showed that the levels of sHVEM were not significantly changed in NSCLC patients upon treatment with nivolumab (Table S1) [90]. However, the same study also reported that patients who responded to nivolumab had lower levels of sHVEM 3 months after treatment than non-responders (Table S3, Fig. 3C) [90]. Further work is needed to explore the role of sHVEM before and after ICI as an indicator of clinical response.

## Baseline soluble immune checkpoints as indicators of clinical response to conventional therapies

Since the levels of soluble immune checkpoints may reflect the immune status of patients, it makes sense that the magnitude of these analytes could correlate with response to ICI, both before and during treatment. However, a large body of literature also indicates that the levels of soluble immune checkpoints, both before and during treatment, correlate with clinical response to non-immunotherapies, or conventional therapies; this may have important implications for combination studies in which ICI is administered in combination with conventional therapies. This section will summarize and discuss studies that have reported that baseline, or pre-treatment, levels of soluble immune checkpoints can indicate clinical response to conventional therapies. For the purposes of this review, conventional therapies refer to non-immunotherapies, and include chemotherapy, targeted drug therapy, radiotherapy, surgery, or combinations of these modalities.

### sPD-L1

**Fig. 4:**
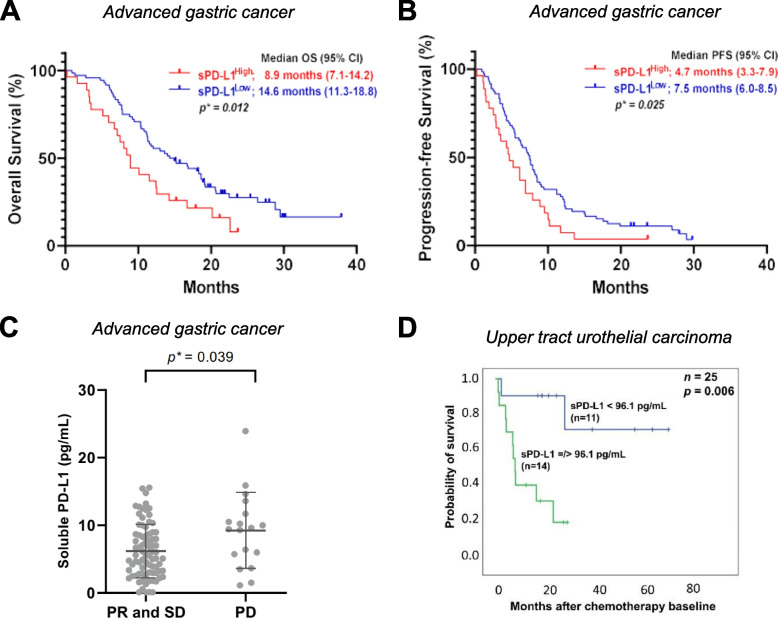
Elevated baseline levels of sPD-L1 correlate with worse response to conventional therapies. Advanced gastric cancer patients treated with chemotherapy were stratified by overall survival (OS) (**A**) and progression-free survival (PFS) (**B**) based on baseline levels of sPD-L1. In this study, levels of sPD-L1 prior to therapy were also significantly lower in those patients developing partial response (PR) or stable disease (SD) after chemotherapy compared to patients developing progressive disease (**C**). Analyses in A and B were performed using the Kaplan–Meier method and the log rank test and in C with an unpaired *t*-test. **D** Upper tract urothelial carcinoma patients treated with chemotherapy were stratified by overall survival based on baseline levels of sPD-L1 ≥ vs < 96.1 pg/mL. PD, progressive disease. Panels (**A**-**C**) modified from Shin, Sci Rep 2023 [218]. Copyright © 2023, Shin et al. Open access license Creative Commons CC BY. Panel (**D**) modified from Szeles, Biomedicines 2022 [89]. © 2022 by Szeles et al. Licensee MDPI, Basel, Switzerland. This article is an open access article distributed under the terms and conditions of the Creative Commons Attribution (CC BY) license (https://creativecommons.org/licenses/by/4.0/)

Elevated baseline sPD-L1 is associated with a poor clinical response to conventional therapies, with 29 different studies involving 3,200 cancer patients reporting a negative association with response rates, PFS or OS (Table 3). Specifically, among cancer patients treated with chemotherapy, higher baseline sPD-L1 levels correlated with shorter OS in those patients with NSCLC [46], gastric cancer [217, 218], pancreatic cancer [219, 220], urothelial cancer [81], and upper tract urothelial carcinoma [89]. As an example, in advanced gastric cancer patients receiving systemic chemotherapy, those with low sPD-L1 prior to therapy had both an improved OS (median: 8.9 months vs 14.6 months, *p* = 0.012, Fig. 4A) and PFS (median: 4.7 months vs 7.5 months, *p* = 0.025, Fig. 4B) compared to patients with higher baseline sPD-L1 [83]. In that study, baseline levels of sPD-L1 also associated with best overall response (BOR), with patients developing SD or PR having significantly lower levels of sPD-L1 prior to therapy than those patients developing progressive disease (PD) (*p* = 0.039, Fig. 4C) [83]. Similarly, patients with upper tract urothelial carcinoma with elevated sPD-L1 levels prior to therapy had a shorter duration of OS (median: ~ 10 months vs not reached at ~ 70 months, *p* = 0.006) following treatment with chemotherapy compared to patients with lower baseline levels (Fig. 4D) [89]. In small cell lung cancer patients receiving chemotherapy consisting of cisplatin-etoposide, elevated levels of sPD-L1 prior to therapy correlated with poor response to therapy and increased rates of death [48].

**Table 3:** Baseline levels of sPD-L1 as an indicator of clinical response to conventional therapies

**Analyte**	**Cancer Type (n)**	**Treatment**	**Material**	**Method**	**Cutoff**	**Association with Clinical Outcome**			
						**Response**	**PFS**	**OS**	**Reference**
↑ sPD-L1	NSCLC (*n* = 109)	Chemotherapy	Serum	ELISA, *ab156361* (Beijing Keyingmei Sci and Tech Ltd.)	0.636 ng/ml			↓ (*p* < 0.001)	[46]
	SCLC (*n* = 250)	Chemotherapy	Serum	ELISA (R&D)	7.0 ng/ml	↓ (*p *= 0.008)			[48]
	Gastric (*n* = 75)	Chemotherapy	Serum	ELISA (USCN)	0.704 ng/ml, 1.081 ng/ml		*ns* *ns*	↓ (*p* = 0.019) ↓ (*p* = 0.0046)	[217]
	Gastric (*n *= 99)	Chemotherapy	Plasma	ELISA, *DY156* (R&D)	9.32 pg/ml	↓ (*p* = 0.039)	↓ (*p* = 0.025)	↓ (*p* = 0.012)	[218]
	Pancreatic ductal adenocarcinoma (*n *= 32)	Chemotherapy	Plasma	ELISA (DYNABIO S.A.)	0.36 ng/ml			↓ (*p* < 0.0001)	[219]
	Pancreatic (*n* = 60)	Chemotherapy	Serum	ELISA, *PDCD1LG1* (USCN)	4.6 ng/ml	*ns*	*ns*	↓ (*p* = 0.015)	[220]
	Urothelial (*n* = 83)	Chemotherapy	Serum	ELISA, *DB7H10* (R&D)	83 pg/ml, 103 pg/ml			↓ (*p* = 0.023) ↓ (*p* = 0.002)	[81]
	Upper tract urothelial (*n* = 25)	Chemotherapy	Serum	ELISA, *DB7H10* (R&D)	96.1 pg/ml, 93.9 pg/ml			↓ (*p* = 0.015)	[89]
	Metastatic gastrointestinal stromal (*n* = 30)	Targeted therapy (imatinib)	Plasma	ELISA (homemade)	0.7 ng/ml		↓ (*p* < 0.0001)		[221]
	Metastatic clear cell renal cell (*n* = 50)	Targeted therapy (sunitinib)	Plasma	ELISA (DYNABIO S.A.)	0.1 ng/ml		↓ (*p* = 0.011)	*ns*	[222]
	NSCLC (*n* = 126)	Radiotherapy	Plasma	ELISA, *PDCD1LG1* (USCN)	96.5 pg/ml			↓ (*p* = 0.005)	[223]
	Gastric (*n* = 180)	Surgery	Serum	ELISA (WLS Cloud-Clone Corp)	0.507 ng/ml		↓ (*p* < 0.0001)	↓ (*p* = 0.0001)	[49]
	Gastric (*n* = 152)	Surgery	Serum	ELISA (R&D)	50 pg/ml			↓ (*p* = 0.02)	[224]
	Gastric (*n* = 116)	Surgery	Serum	ELISA (R&D)	57 pg/ml		↓ (*p* = 0.025)	*ns*	[74]
	Hepatocellular (*n* = 120)	Surgery	Serum	hIC antibody array assay, *QAH-ICM-1–1* (RayBiotech)	11.2 μg/ml		↓ (*p* = 0.023)	↓ (*p* = 0.048)	[225]
	Colorectal (*n* = 131)	Surgery	Serum	ELISA (WLS Cloud-Clone Corp)	0.08 ng/ml		↓ (*p* = 0.05)	↓ (*p* = 0.01)	[226]
	Colorectal with liver metastasis (*n* = 177)	Surgery	Plasma	ELISA (R&D)	551.82 pg/ml		↓ (*p* = 0.0041)	↓ (*p* = 0.0061)	[227]
	Renal cell (*n* = 144)	Surgery	Serum	ELISA, *DB7H10* (R&D)	87.2 pg/ml			↓ (*p* = 0.002)	[72]
	Upper tract urothelial (*n* = 37)	Surgery	Serum	ELISA, *DB7H10* (R&D)	84.0 pg/ml, 118.5 pg/ml			↓ (*p* = 0.041) ↓ (*p* < 0.001)	[89]
	Head and neck (*n* = 60)	Surgery	Serum	ELISA (Sunred Bio)	0.765 ng/ml		↓ (*p* = 0.035)	*ns*	[228]
	Hepatocellular (*n* = 53)	Radiotherapy or chemo-radiotherapy	Plasma	ELISA (R&D)	1.315 pg/ml			↓ (*p* = 0.037)	[71]
	Hepatocellular (*n* = 215)	Resection, local ablation, sorafenib or liver transplantation	Serum	ELISA, *PDCD1LG1* (USCN)	0.8 ng/ml			↓ (*p* < 0.001)	[69]
	Hepatitus B-related hepatocellular (*n* = 81)	Surgery or thermal ablation	Serum	ELISA (USCN)	2.825 ng/ml		↓ (*p* = 0.002)	↓ (*p* = 0.012)	[52]
	Renal cell (*n* = 181)	Surgery, surgery and sunitinib, or surgery and axitinib	Serum	ELISA, *CSB-E13644h* (Cusabio Biotech)	18.3 pg/ml			↓ (*p* < 0.00001)	[58]
	Clear cell renal cell (*n* = 89)	Surgery with or without systemic therapy	Plasma	DuoSet ELISA, *DY156* (R&D)	793 ng/ml			↓ (*p* = 0.0001)	[70]
	Nasopharyngeal (*n* = 219)	Radiotherapy or chemo-radiotherapy	Plasma	ELISA, *ab214565* (Abcam)	93.7 pg/ml		↓ (*p* = 0.006)		[55]
	Soft tissue sarcoma (*n* = 86)	Surgery or radiotherapy	Serum	ELISA, *ab214565* (Abcam)	44.26 pg/ml		↓ (*p* < 0.001)	↓ (*p* = 0.011)	[229]
	Ovarian (*n* = 37)	Surgery or neoadjuvant chemotherapy	Serum	hIO Checkpoint Protein Magnetic Bead Panel 1 (Merck Millipore)	0.30 pg/ml		↓ (*p* = 0.016)	↓ (*p* = 0.048)	[23]
	Epithelial ovarian (*n* = 83)	Surgery and chemotherapy	Serum	ELISA (R&D)	6.4 pg/ml		↓ (*p* = 0.019)	↓ (*p* = 0.003)	[60]
	High-grade serous ovarian (*n* = 100)	Surgery and chemotherapy	Plasma	ELISA (DYNABIO S.A.)	0.42 ng/ml		↓ (*p* < 0.0001)		[230]

*Abbreviations: PFS* Progression-free survival, *OS* Overall survival, *hIC* Human immune checkpoint, *hIO* Human Immuno-Oncology, *NSCLC* Non-small cell lung cancer, *SCLC* Small cell lung cancer, *ns* Not significant, *sPD-L1* Soluble programmed cell death-ligand 1

In patients receiving targeted drug therapy, high baseline sPD-L1 associated with shorter PFS in patients with metastatic gastrointestinal stromal tumors [221] and clear cell renal cell carcinoma [222]. Among cancer patients treated with radiotherapy, elevated baseline sPD-L1 correlated with shorter OS in patients with NSCLC [223] and hepatocellular carcinoma [71]. High baseline sPD-L1 was also correlated with worse distant metastasis-free survival (74.0% vs 87.5% rate at 4 years) after radiotherapy or chemo-radiotherapy in nasopharyngeal carcinoma patients [55].

Many studies have also found a negative association between elevated pre-treatment sPD-L1 and response of cancer patients to surgery (Table 3). Higher baseline sPD-L1 correlated with shorter OS after surgical resection in patients with gastric cancer [49, 224], colorectal cancer [226, 227], hepatocellular carcinoma [225], renal cell carcinoma [72], soft tissue sarcoma [229], and upper tract urothelial carcinoma [89]. Elevated sPD-L1 prior to surgery was also associated with worse disease-free survival (DFS) in patients with gastric cancer [49], colorectal cancer [226], head and neck cancer [228], and hepatocellular carcinoma [225] and with worse relapse-free survival in patients with gastric cancer [74] and colorectal cancer with liver metastasis [227]. Elevated pre-treatment sPD-L1 levels were also associated with lower metastasis-free survival (42.4% vs 88.4% rate at 5 years) following surgery in patients with soft tissue sarcoma [229]. Among patients with hepatitis B virus (HBV)-related hepatocellular carcinoma, elevated baseline sPD-L1 correlated with shorter OS and DFS following treatment with surgery or thermal (radiofrequency or microwave) ablation [52]. High baseline sPD-L1 was also correlated with shorter OS and PFS in ovarian cancer patients receiving surgery or neoadjuvant chemotherapy [23] and with shorter OS in hepatocellular carcinoma patients treated with resection, local ablation, sorafenib or liver transplantation [69].

There are also several studies reporting on the association between baseline levels of sPD-L1 and response to combination treatment with conventional therapies (Table 3). In all cases, elevated baseline sPD-L1 correlated with worse clinical outcomes. Among renal cell carcinoma patients, high pre-treatment sPD-L1 correlated with shorter OS after treatment with (1) surgery, (2) surgery and first-line treatment with sunitinib, or (3) surgery and second-line treatment with axitinib [58]. In patients with clear cell renal cell carcinoma, high baseline sPD-L1 correlated with worse 5-year OS rate after surgery in non-metastatic patients and a combination of nephrectomy and systemic therapy in metastatic patients [70]. Among epithelial ovarian cancer patients, high baseline sPD-L1 correlated with reduced PFS and OS 5 years after treatment with surgery and chemotherapy [60]. Elevated baseline sPD-L1 also correlated with shorter PFS (median: 24 vs 40 months) after combination treatment with surgery and chemotherapy in patients with high-grade serous ovarian cancer [230].

While most studies show that elevated baseline sPD-L1 is associated with a worse clinical response to a variety of conventional therapies, a few studies (*n* = 8) have reported baseline sPD-L1 to have either a negligible [51, 62, 222, 231–233] or positive [47, 234] prognostic value. Baseline sPD-L1 levels were not significantly associated with clinical outcomes in pancreatic cancer patients treated with chemotherapy [231], NSCLC patients receiving chemotherapy or targeted drug therapy [62] or treated with surgery [233], hepatocellular carcinoma patients treated with radiotherapy [232] or transarterial chemoembolization (TACE) [51], and metastatic clear cell renal cell carcinoma patients receiving bevacizumab [222]. sPD-L1 levels at baseline were positively associated with clinical outcome in NSCLC patients after surgery [47] and hepatocellular carcinoma patients following hepatic resection or liver transplantation [234]. Taken all together, most studies demonstrate that elevated circulating sPD-L1 prior to treatment with chemotherapy, radiotherapy, targeted drug therapy or surgery is associated with a poor clinical response to these conventional therapies.

### sPD-1

**Fig. 5:**
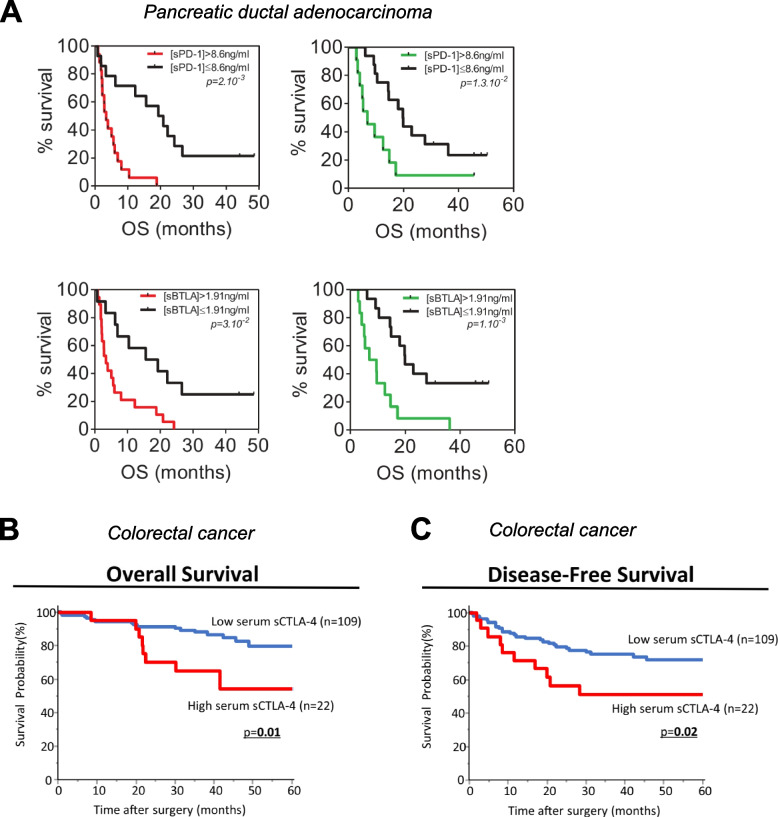
Elevated baseline levels of soluble immune checkpoints correlate with worse response to conventional therapies. Kaplan-Meier analysis of overall survival (OS) in patients with pancreatic ductal adenocarcinoma treated with chemotherapy stratified by baseline levels of sPD-1 and sBTLA (**A**) in a learning cohort (left) and validation cohort (right). Levels of sCTLA4 prior to therapy in colorectal cancer patients treated with surgery associated with both OS (**B**) and disease-free survival (**C**). Panel (**A**) modified from Bian, OncoImmunology, 2019 [219]. Reprinted by permission of the publisher Taylor & Francis Ltd., http://www.tandfonline.com. Panels (**B** and **C**) modified from Omura, Cancer Immunol Immunother 2020 [226]. Reproduced with permission from Springer Nature, https://www.springernature.com/gp

While there are certainly fewer studies focusing on circulating levels of sPD-1 than sPD-L1, six different reports involving 299 cancer patients have shown that high levels of sPD-1 prior to conventional therapy correlated with a shorter PFS or OS following treatment (Table 4). Specifically among patients treated with chemotherapy, higher baseline sPD-1 levels were correlated with shorter OS (median: 3.4 vs 20.0 months) in patients with pancreatic adenocarcinoma (Fig. 5A) [219]. In addition, in patients receiving targeted drug therapy, high baseline sPD-1 associated with shorter PFS in patients with metastatic gastrointestinal stromal tumors [221] and clear cell renal cell carcinoma [222]. In patients with ovarian cancer, elevated baseline sPD-1 associated with a shorter 5-year OS rate (median: 37 vs 49 months) in patients treated with surgery [24], and a shorter duration of PFS in patients treated with surgery or neoadjuvant chemotherapy [23]. Furthermore, among patients with high-grade serous ovarian cancer, elevated baseline sPD-1 correlated with shorter PFS (median: 24 vs 30 months) following combination treatment with surgery and chemotherapy [230].

**Table 4:** Baseline levels of other soluble immune checkpoints as indicators of clinical response to conventional therapies

**Analyte**	**Cancer Type (n)**	**Treatment**	**Material**	**Method**	**Cutoff**	**Association with Clinical Outcome**			
						**Response**	**PFS**	**OS**	**Reference**
↑ sPD-1	Pancreatic ductal adenocarcinoma (*n* = 32)	Chemotherapy	Plasma	ELISA (DYNABIO S.A.)	8.6 ng/ml			↓ (*p* = 0.002)	[219]
	Metastatic gastrointestinal stromal (*n* = 30)	Targeted therapy (imatinib)	Plasma	ELISA (homemade)	8.1 ng/ml		↓ (*p* = 0.0001)		[221]
	Metastatic clear cell renal cell (*n* = 50)	Targeted therapy (sunitinib)	Plasma	ELISA (DYNABIO S.A.)	1.67 ng/ml		↓ (*p* = 0.009)	*ns*	[222]
	Ovarian (*n* = 50)	Surgery	Plasma	ELISA (Biorbyt LLC.)	75.06 pg/ml			↓ (*p* < 0.05)	[24]
	Ovarian (*n* = 37)	Surgery or neoadjuvant chemotherapy	Serum	hIO Checkpoint Protein Magnetic Bead Panel 1 (Merck Millipore)	3.33 pg/ml		↓ (*p* = 0.035)	*ns*	[23]
	High-grade serous ovarian (*n* = 100)	Surgery and chemotherapy	Plasma	ELISA (DYNABIO S.A.)	2.48 ng/ml		↓ (*p* = 0.02)		[230]
↑ sCTLA4	Colorectal (*n* = 131)	Surgery	Serum	ELISA (Life Technologies)	1.79 ng/ml		↓ (*p* = 0.02)	↓ (*p* = 0.01)	[226]
	Chronic hepatitis C-hepatocellular (*n* = 88)	Radiofrequency ablation	Serum	ELISA	9 ng/ml		↓ (*p* = 0.017)		[126]
	Prostate (*n* = 190)	Surgery, radiotherapy or surveillance	Serum	ProcartaPlex hIO Checkpoint Panel (Thermo Fisher)	89.28 pg/ml		↓ (*p* = 0.010)		[235]
↑ sCD80	Soft tissue sarcoma (*n* = 85)	Surgery	Serum	ELISA (Abcam)	404 pg/ml OS, 531 pg/ml MFS		↓ (*p* = 0.016)	↓ (*p* = 0.015)	[138]
	Prostate (*n* = 190)	Surgery, radiotherapy or surveillance	Serum	ProcartaPlex hIO Checkpoint Panel (Thermo Fisher)	51.24 pg/ml		↓ (*p* = 0.030)		[235]
↑ sTIM3	Clear cell renal cell (*n* = 182)	Surgery or a combination of surgery and chemotherapy	Plasma	Procarta-Plex hIO Checkpoint Panel (Thermo Fisher)	5908 pg/ml			↓ (*p* = 0.0039)	[153]
	Osteosarcoma (*n* = 120)	Neoadjuvant chemotherapy, radical surgery, and chemotherapy	Serum	ELISA (Sigma)	14.4 ng/ml			↓ (*p* < 0.01)	[151]
↑ sLAG3	Lung (*n* = 83)	Surgery, surgery and chemotherapy, surgery and radiotherapy, or surgery and immunotherapy	Plasma	LegendPlex Custom Human Immune Checkpoint Panel (Biolegend)	722.5 pg/ml		↓ (*p* = 0.0003)		[236]
	Hepatocellular (*n* = 100)	TACE	Serum	ELISA, *BMS2211* (Thermo Fisher Scientific)	3723.1 pg/ml	↓ (*p* = 0.002)		↓ (*p* < 0.001)	[51]
	Advanced head and neck (*n* = 23)	chemotherapy or αPD-1	Serum	hIO Checkpoint 14-plex ProcartaPlex Panel 1, *EPX14A-15803–901* (Thermo Fisher Scientific)	377 pg/ml		↓ (*p* = 0.047)	↓ (*p* = 0.001)	[176]
↑ sB7-H3	Hepatocellular (*n* = 149)	Surgery	Serum	ELISA (R&D)	48.34 ng/ml			↓ (*p* = 0.037)	[191]
	Non-muscle-invasive bladder (*n* = 555)	Surgery	Serum	ELISA (LifeSpan BioScience)	0 ng/ml		↓ (*p* = 0.0002)		[192]
↑ sBTLA	Pancreatic ductal adenocarcinoma (*n* = 32)	Chemotherapy	Plasma	ELISA (DYNABIO S.A.)	1.91 ng/ml			↓ (*p* = 0.03)	[219]
	Hepatocellular (*n* = 53)	Targeted therapy (sorafenib)	Plasma	Multiplexed immunoassays with the Milliplex Map Kit (EMD Millipore)	395 pg/ml			↓ (*p* = 0.038)	[237]
	Clear cell renal cell (*n* = 182)	Surgery or a combination of surgery and chemotherapy	Plasma	Procarta-Plex hIO Checkpoint Panel (Thermo Fisher)	2269 pg/ml			↓ (*p* = 0.00139)	[153]
	Prostate (*n* = 190)	Surgery, radiotherapy or surveillance	Serum	ProcartaPlex hIO Checkpoint Panel (Thermo Fisher)	506.56 pg/ml		↓ (*p* = 0.003)		[235]
	High-grade serous ovarian (*n* = 100)	Surgery and chemotherapy	Plasma	ELISA (DYNABIO S.A.)	2.78 ng/ml		↓ (*p* = 0.0002)		[230]
↑ sHVEM	Prostate (*n* = 190)	Surgery, radiotherapy or surveillance	Serum	ProcartaPlex hIO Checkpoint Panel (Thermo Fisher)	29.00 pg/ml		↓ (*p* = 0.007)		[235]

*Abbreviations: PFS* Progression-free survival, *OS* Overall survival, *hIO* Human Immuno-Oncology, *sPD-1* Soluble programmed cell death protein 1, *ns* Not significant, *sCTLA4* Soluble cytotoxic T-lymphocyte-associated antigen 4, *sCD80* Soluble CD80, *sTIM3* Soluble T cell immunoglobulin and mucin domain-containing protein 3, *sLAG3* Soluble lymphocyte activation gene 3, *sB7-H3* Soluble B7-H3, *sBTLA* Soluble B and T lymphocyte attenuator, *sHVEM* Soluble herpesvirus entry mediator, *TACE* transarterial chemoembolization

Four studies involving 198 patients have found baseline sPD-1 to have a negligible [63, 222, 231, 232] prognostic value in cancer patients treated with conventional therapies. Specifically, baseline levels of sPD-1 did not associate with clinical outcomes in esophageal cancer patients receiving chemotherapy (with or without radiation or surgical resection) [63], pancreatic cancer patients treated with chemotherapy [231], metastatic clear cell renal cell carcinoma receiving bevacizumab [222], and hepatocellular carcinoma patients treated with radiotherapy [232]. Conversely, a single study found that high baseline sPD-1 correlated with longer DFS and OS in hepatocellular carcinoma patients after surgical resection [225]. Collectively, these studies demonstrate that high sPD-1 levels at baseline, in some cases but not others, associate with poor clinical outcomes following treatment with conventional therapies. These discrepant findings may be impacted by multiple factors, including the specific type, dose, and schedule of the conventional therapy evaluated.

### sCTLA4

Three studies involving 409 patients with solid tumors have evaluated the association between baseline levels of sCTLA4 and response to conventional therapies, with all reporting a negative association with PFS (Table 4). In colorectal cancer patients treated with surgery, higher baseline sCTLA4 levels correlated with both shorter OS (Fig. 5B) and DFS (Fig. 5C) [226]. Elevated baseline sCTLA4 also correlated with a higher risk of biochemical recurrence and increased progression in prostate cancer patients after treatment with radical prostatectomy, radiotherapy or surveillance [235]. Finally, among patients with hepatocellular carcinoma and chronic hepatitis C, high baseline sCTLA4 correlated with earlier local recurrence and development of intrahepatic metastasis after treatment with radiofrequency ablation [126]. Collectively these studies demonstrate that elevated levels of sCTLA4 prior to therapy associate with poor outcomes in cancer patients treated with a variety of conventional therapies.

### sCD80, sTIM3, sLAG3, sB7-H3, sBTLA and sHVEM

While the soluble immune checkpoints sCD80, sTIM3, sLAG3, sB7-H3, sBTLA, and sHVEM are far less studied than sPD-L1, sPD-1, and sCTLA4, elevated levels are generally associated with a poor clinical response to a variety of conventional therapies. A total of 12 different studies involving 1,672 patients have reported that high levels at baseline of at least one of these analytes (sCD80, sTIM3, sLAG3, sB7-H3, sBTLA, and sHVEM) are associated with poor clinical outcomes following conventional therapy (Table 4). Higher baseline sBTLA levels correlated with shorter OS in pancreatic adenocarcinoma patients receiving chemotherapy (median: 3.4 vs 17.4 months, Fig. 5A) [219], and in hepatocellular carcinoma patients after treatment with sorafenib, a targeted drug therapy (median: 8.4 vs 20.3 months) [237]. High baseline sLAG3 associated with poor ORR (lower frequencies of CR and PR), and shorter OS (median: 13.63 vs 34.43 months) in hepatocellular carcinoma patients treated with TACE [51]. In patients having had surgical resection, high baseline sCD80 associated with lower metastasis-free survival (44.0% vs 75.3% rate at 5 years) and shorter OS (65.0% vs 89.5% rate at 5-years) in soft tissue sarcoma patients [138], while high baseline sB7-H3 correlated with lower OS (median: 25.62 vs 47.75 months) in hepatocellular carcinoma patients [191]. High baseline sB7-H3 correlated with shorter recurrence-free survival (25.4% vs 60.2% rate at 3-years and 23.2% vs 51.9% rate at 5 years) and PFS (85.0% vs 95.0% rate at 3 years and 68.8% vs 91.7% rate at 5-years) in non-muscle-invasive bladder cancer patients treated with transurethral resection [192]. In addition, in prostate cancer patients treated with radical prostatectomy, radiotherapy or surveillance, high baseline levels of both sCD80 and sHVEM correlated with a higher risk of biochemical recurrence and increased progression, while high baseline sTIM3 and sBTLA correlated with increased aggressiveness [235]. In that study, high baseline sBTLA also correlated with an increased rate of disease progression. High baseline sLAG3 associated with both lower PFS and OS in advanced head and neck cancer patients after treatment with chemotherapy [176].

In clear cell renal cell carcinoma patients treated with surgery or a combination of surgery and chemotherapy, high baseline sTIM3 and sBTLA associated with reduced OS (Table 4) [153]. In addition, high pre-treatment sTIM3 correlated with lower OS in osteosarcoma patients after treatment with neoadjuvant chemotherapy, radical surgery, and chemotherapy [151]. Among patients with high-grade serous ovarian cancer, elevated baseline sBTLA correlated with shorter PFS (median: 24 vs 32 months) following surgery and chemotherapy [230]. Finally, high baseline sLAG3 was associated with tumor relapse and shorter relapse-free survival in lung cancer patients after treatment with surgical resection alone or in combination with either chemotherapy or radiotherapy [236]. Only two studies to date have found any of these soluble checkpoints at baseline to have either a negligible [152, 238] or positive [238] prognostic value.

## Post-treatment levels of soluble immune checkpoints after conventional therapies as indicators of clinical response

### sPD-L1

As observed with ICI, plasma and serum levels of soluble immune checkpoints can change upon treatment with conventional therapies and these changes can, in some cases, correlate with clinical response. Numerous studies (*n* = 22, involving 1,223 patients) have reported on the effect of conventional therapies, including chemotherapy, targeted drug therapy, radiotherapy, surgery, or combinations of these modalities, on the level of sPD-L1 (Table S4). The majority of these studies have found that sPD-L1 is increased in patients upon treatment [62, 65, 67, 71, 89, 232, 233, 237, 239–243]; however, others have reported that levels either remained constant [51, 62, 64, 68, 81, 89, 222, 243] or decreased [55, 223, 227, 244] following treatment with conventional therapies.

**Fig. 6:**
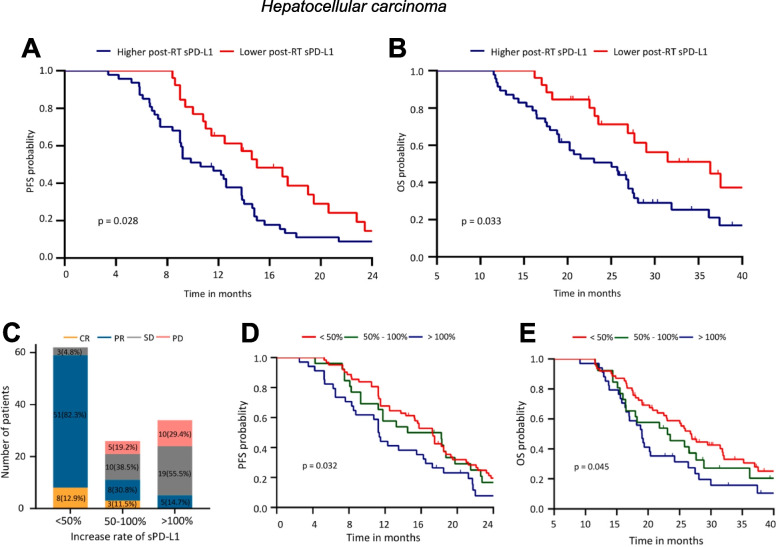
Post treatment levels of sPD-L1 after conventional therapy associate with patient response. Kaplan–Meier analyses of (**A**) progression-free survival (PFS) and (**B**) overall survival (OS) in hepatocellular carcinoma patients with sPD-L1 levels > vs < 14.60 pg/ml after treatment with radiotherapy. The degree of change in sPD-L1 after radiotherapy compared to baseline also associated with overall response rate (**C**), PFS (**D**), and OS (**E**). Panels (**A**-**E**) from Zhang, Transl Oncol, 2022 [232]. Copyright © 2022. Published by Elsevier Inc

Many fewer studies (*n* = 8, involving 513 patients) have reported on the association between patient outcomes and levels or changes in the levels of sPD-L1 following treatment with conventional therapies (Table 5). In general, as was seen with ICI, a decrease or less of an increase in sPD-L1 upon treatment with conventional therapy is typically correlated with better response, while greater increases or higher levels are often associated with poor clinical responses. Pancreatic cancer patients who responded to chemotherapy experienced a reduction in sPD-L1 after three cycles of treatment; 48.3% of patients who experienced a decrease in sPD-L1 achieved CR or PR, compared to only 20.8% of patients with stable levels or increases in sPD-L1 [220]. Among triple-negative breast cancer patients receiving neoadjuvant chemotherapy, those who achieved CR or PR had reduced sPD-L1 after treatment, while non-responders (with either SD or progressive disease (PD)) had no change after treatment [64]. In addition, patients with locally advanced rectal cancer who responded to neoadjuvant chemoradiotherapy experienced decreases in sPD-L1 during the course of treatment with sPD-L1 that returned to baseline levels by the end of treatment; non-responders, in contrast, experienced no change in sPD-L1 throughout the course of treatment [244]. Metastatic renal cell carcinoma patients who were responsive to sunitinib or pazopanib (both targeted therapies) had lower levels of sPD-L1 after 3–4 months of treatment (56.25 pg/mL) than non-responders (146.5 pg/mL) [245]. Furthermore, locally advanced rectal cancer patients treated with neoadjuvant chemoradiotherapy experienced a correlation between high sPD-L1 after treatment and an increased presence of lymphovascular invasion [239], while high sPD-L1 after hepatic resection in colorectal cancer patients with liver metastasis was indicative of a higher early recurrence rate (52.9% vs 13.8%) and shorter relapse-free survival (median of 5.87 vs 15.54 years, *p* = 0.0041) [227]. Lastly, in patients with hepatocellular carcinoma, high sPD-L1 levels after radiotherapy identified patients with significantly shorter PFS (median: 13.25 vs 18.75 months, *p* = 0.028, Fig. 6A) and OS (median: 25.03 vs 36.33 months, *p* = 0.033, Fig. 6B) [232]. In this study, not only higher post treatment levels, but also a higher rate of increase in sPD-L1 after radiotherapy correlated with a worse response rate (lower frequency of CR and PR, Fig. 6C) and a shorter duration of PFS (*p* = 0.032, Fig. 6D) and OS (*p* = 0.045, Fig. 6E) [232]. Only one study, in hepatocellular carcinoma patients treated with sorafenib, found no association between changes in sPD-L1 and patient outcome [237]. Collectively, these studies indicate that an increase in sPD-L1 after conventional therapy associates with poor clinical response.

**Table 5:** Post-treatment levels of sPD-L1 after conventional therapies as an indicator of clinical response

**Analyte**	**Cancer Type (n)**	**Treatment**	**Material**	**Method**	**Direction Post- treatment**	**Association with Clinical Outcome**			
						**Response**	**PFS**	**OS**	**Reference**
sPD-L1	Pancreatic (*n* = 60)	Chemotherapy	Serum	ELISA, *PDCD1LG1* (USCN)	↓	↑ (*p* = 0.038)			[220]
	Triple-negative breast (*n* = 66)	Neoadjuvant chemotherapy	Serum	ELISA (Jianglai Biological)	↓	↑ (*p* = 0.021)			[64]
	Metastatic renal cell carcinoma (*n* = 20)	Targeted drug therapy (sunitinib or pazopanib) as first-line therapy	Serum	hIO Checkpoint 14-Plex ProcartaPlex Panel 1, *EPX14A-15803–901* (eBioscience)	↓	↑ (*p* = 0.03)			[245]
	Hepatocellular (*n* = 122)	Radiotherapy	Serum	hIO Checkpoint Protein Panel, *CHCKPMAG-11 K* (Merck)	↓	↑ (*p* < 0.001)	↑ (*p* = 0.032)	↑ (*p* = 0.045)	[232]
	Colorectal with liver metastasis (*n* = 49)	Surgery	Plasma	ELISA (R&D)	↓	↑ (*p* = 0.011)	↑ (*p* = 0.0041)		[227]
	Locally advanced rectal (*n* = 30)	Neoadjuvant chemoradiotherapy	Plasma	MILLIPLEX® MAP hIO Checkpoint Protein Panel, *HCKPMAG-11 K* (Millipore Sigma)	↓	↑ (*p* < 0.05)			[244]
	Locally advanced rectal cancer (*n* = 113)	Neoadjuvant chemoradiotherapy	Serum	ELISA, *SEA788Hu* (Cloud-Clone Corp)	↓	↑ *(p* = *0.0752)*			[239]

*Abbreviations: PFS* Progression-free survival, *OS* Overall survival, *hIO* Human Immuno-Oncology, *sPD-L1* Soluble programmed cell death-ligand 1

### sPD-1

Multiple studies (*n* = 13, involving 641 patients) have also reported on the effects of conventional therapies on the level of sPD-1, with some reporting an increase [65, 68, 237, 240, 246], no change [64, 67, 222, 239, 241, 243] or a decrease [242, 245] after therapy (Table S5). Only four studies, involving 187 patients, have evaluated the association between levels or changes in the levels of sPD-1 after conventional therapy and patient outcome (Table S6). One study found that triple-negative breast cancer patients developing CR or PR had reduced sPD-1 after treatment with neoadjuvant chemotherapy, while non-responders (with SD or PD) had no change after treatment [64]. In contrast, an increase in sPD-1 after conventional treatment with erlotinib was correlated with longer PFS and OS in NSCLC patients [246], and lower levels of sPD-1 post-surgery were associated with worse rate of OS at 2 years (60% vs 93%) in patients with gastric carcinoma [68]. Finally, one study also reported that the change in sPD-1 following 2 weeks of sorafenib had no association with response to therapy in hepatocellular carcinoma patients [237]. These studies highlight that sPD-1 levels or changes in levels after conventional therapy do not consistently associate with patient outcomes, and further work is needed to understand its relevance in this setting.

### sCTLA4

Eight studies comprising 446 patients with solid malignancies have reported on changes in levels of sCTLA4 following conventional treatments (Table S5). While most showed that sCTLA4 is increased [126, 237, 240, 242, 243, 247], several studies have found that sCTLA4 either did not change [65, 243] or was reduced [67] following treatment with various conventional therapies. Only four studies with a total of 249 patients have reported on the relationship between levels or changes in levels sCTLA4 following treatment with conventional therapies and clinical response, and with conflicting findings (Table S6). Metastatic renal cell carcinoma patients who were responsive to sunitinib or pazopanib had lower levels of sCTLA4 after 3–4 months of treatment (281.6 pg/mL) than non-responders (616.4 pg/mL) [245]. Similarly, an increase in sCTLA4 3 days after treatment with radiofrequency ablation was only observed in chronic hepatitis C-hepatocellular carcinoma patients who exhibited early recurrence, while levels in patients without early recurrence remained constant [126]. In contrast, high levels of sCTLA4 after radiotherapy, chemotherapy, and/or chemoradiotherapy correlated with improved outcomes including longer OS and PFS in patients with lung, esophageal, liver, ovarian or cervical cancer [247]. Finally, one study found that changes in sCTLA4 after 2 weeks of sorafenib treatment had no association with response to therapy in hepatocellular carcinoma patients [237]. These studies highlight that sCTLA4 levels or changes in levels after conventional therapy do not consistently associate with patient response and further studies are needed to evaluate this association.

### sCD80, sTIM3, sLAG3, sBTLA and sHVEM

Eleven different studies (involving 405 patients) have also evaluated the levels of sCD80, sTIM3, sLAG3, sBTLA, or sHVEM following treatment with conventional therapies (Tables S7 and S8). In some cases, some of these analytes increased [65, 67, 152, 237, 240, 242, 243], while in other cases reductions [51, 56, 65, 67, 237, 242, 244, 245] or no changes [65, 240, 243] were noted. Few studies (*n* = 5 and involving 189 patients) have evaluated the association between levels and/or changes in levels of sCD80, sTIM3, sLAG3, sBTLA, and sHVEM after conventional therapy and clinical outcome (Table S6). Some studies found that lower levels of these analytes after starting conventional therapy associate with improved outcome. For example, locally advanced cervical cancer patients developing a CR following concurrent chemoradiotherapy had lower levels of sLAG3 after therapy than those with PR or SD [240]. Similarly, hepatocellular carcinoma patients who responded to TACE had lower sLAG3 levels 3 days after treatment than did non-responders [51]. Contrasting findings have also been reported; Tampaki et al. found that hepatocellular carcinoma patients with CR following TACE treatment had higher sTIM3 levels 1 week after treatment than those who went on to develop a PR (median: 534 vs 222 pg/mL) [152]. Furthermore, locally advanced rectal cancer patients who responded to neoadjuvant chemoradiotherapy showed decreased levels of sCD80 during treatment that increased to baseline after treatment ended; poor responders also had decreased levels of sCD80 during treatment; however, the level of sCD80 remained low in these patients following cessation of treatment [244]. Finally, one study in hepatocellular carcinoma patients found that changes in the levels of sCD80, sTIM3, sLAG3, sBTLA, and sHVEM following 2 weeks of sorafenib treatment had no association with response to therapy [237]. Overall, these studies, like those with sPD-1 and CTLA4, highlight that changes in sCD80, sTIM3, sLAG3, sBTLA, or sHVEM do not consistently associate with patient response following conventional therapy.

## Conclusions and future directions

ICI have revolutionized cancer immunotherapy. The concept of manipulating the immune system to recognize and target tumor antigens is extremely promising and has led to improved clinical benefit across multiple tumor indications [6–9, 11]. However, many patients remain resistant to this modality of treatment [14, 15], prompting the need to identify relevant biomarkers to predict response. Blood-based biomarkers are appealing for this purpose, due to technical and practical advantages over traditional tumor biopsies [1–3]. It should be noted that peripheral immune analyses can complement analyses of tumor biopsies and should be paired together whenever practically possible. Soluble immune checkpoints seem likely to be relevant in patients treated with both ICI and conventional therapies.

All the soluble checkpoints discussed here can bind to the same ligands as their membrane-bound counterparts, thus having the potential to modulate cytokine secretion, T cell viability, and T cell proliferation. Overall, sPD-L1, sCTLA4, sB7-H3, and sHVEM have primarily immunosuppressive functions, while sLAG3 and sBTLA have immunostimulatory functions, and sPD-1, sCD80, and sTIM3 are not as well functionally defined. Most of these soluble immune checkpoints (with the exception of sCD80) are elevated in cancer patients compared to healthy donors, with higher levels correlating with a more advanced/aggressive stage of cancer. This information may have diagnostic implications, as elevated levels may indicate the presence and/or stage of disease, or at the very least provide rationale for further diagnostic testing. In addition, many studies reported a prognostic value of these soluble immune checkpoints as indicators of clinical response to both ICI and conventional therapies. Numerous studies have shown that elevated levels of sPD-L1 prior to treatment are associated with worse response to ICI, while the other soluble immune checkpoints discussed require further investigation to determine their correlation with response. Greater increases and/or elevated levels of sPD-L1 and sPD-1 after ICI are associated with poor clinical outcome, while the literature is scarce and more conflicting regarding this association for the other soluble immune checkpoints reviewed here.

Surprisingly, a large body of literature has observed correlations between levels of soluble immune checkpoints in cancer patients and response to conventional, non-immunotherapies. This may support an indirect role of the immune system in the clinical response of patients to conventional therapies. Most studies indicated that elevated levels of the soluble immune checkpoints reviewed (sPD-L1, sPD-1, sCTLA4, sCD80, sTIM3, sLAG3, sB7-H3, sBTLA, and sHVEM) prior to conventional therapies correlated with poor clinical response. Most of these analytes were also increased following conventional therapies. However, with the exception of sPD-L1, changes observed in soluble immune checkpoints following conventional therapies have an unclear prognostic value. With sPD-L1, greater increases and/or elevated levels after conventional treatments are associated with poor clinical response to conventional therapy. These findings support the continued investigation of soluble immune checkpoints as potential biomarkers of response and may aid in the development of combination therapies.

Several gaps remain regarding the role that soluble immune checkpoints play in modulating an anti-tumor response. First, the exact functions of certain soluble immune checkpoints remain unclear. sPD-L1, the most well-studied among the soluble immune checkpoints, clearly has immunosuppressive functions [35, 36, 38, 41–43]. However, functional studies of sPD-1, sCD80, and sTIM3 are conflicting and inconsistent. Next, studies have shown that these soluble immune checkpoints can bind to the same ligands as their membrane-bound counterparts. Further work is needed to understand whether this serves to enhance immune suppressive signaling (as appears to be the case for sPD-L1) or block immune suppressive signaling, as has been proposed for sTIM3. In addition, in some cases, the function of the soluble immune checkpoint does not appear to align with what is known about its prognostic value. For example, extensive studies have shown sLAG3 to have anti-tumor functions [163–172]. However, higher baseline levels and an increase in sLAG3 correlate with a worse clinical response following both ICI and conventional therapy. This same disconnect between function and association with clinical outcome is seen with sBTLA. One reason for the disconnect may be that the functional studies were mostly conducted in vitro and in mice, while prognostic studies were performed in humans. It is also unknown whether variations in the levels of soluble immune checkpoints are reflective of the tumor burden, underlying immune activity, immune response to therapy, or a combination of these aspects. Finally, very little is known regarding the regulation of the production of these soluble immune checkpoints. Few studies have compared the expression of membrane-bound immune checkpoints with their soluble counterparts. In the case of PD-L1, there was no correlation in patients with various solid tumors between membrane-bound tumor expression of PD-L1 and circulating sPD-L1 levels [49, 82, 85, 86, 225, 226, 229, 234]. Additional studies are needed to investigate possible associations between the expression of other membrane-bound immune checkpoints and their soluble counterparts. Studies should also be performed to investigate whether certain physiological conditions can regulate the cleavage of membrane-bound immune checkpoints to induce the soluble forms, and whether certain conditions may halt this production process.

In addition to these theoretical questions, there are also practical issues that must be considered to implement the use of soluble immune checkpoints as biomarkers of clinical response to therapy. Many variables exist among the studies reviewed, such as the material measured (serum or plasma), the assay used (ELISA or multiplex assay), variation in timepoints in which samples were collected with respect to a given treatment, and a high degree of variation in the cutoff values of soluble immune checkpoints that were used to stratify patients into “high” vs “low” groups. In addition to a range of values, the method of obtaining these cutoffs also varied, with some studies using the mean, median, or 25th percentile or 75th percentile, while others determined cutoffs through receiver operating characteristic (ROC) curves, maximally selected log rank statistics, or spline curve analyses. Ideally, the method of cutoff determination and sample measurement will be optimized and standardized before widespread use. It should be noted that while most studies used ELISAs to measure sPD-L1 and sPD-1, the other soluble immune checkpoints reviewed here (sCTLA4, sCD80, sTIM3, sLAG3, sB7-H3, sBTLA, and sHVEM) were typically quantified through multiplexed immunoassays that screened for a panel of analytes. The use of common platforms, well-established analytical methods, and prospective evaluation in large clinical studies with well-defined clinical endpoints will be necessary to advance the utility of soluble immune checkpoints as a biomarker to guide treatment decisions. In addition, controlled studies directly comparing the performance of soluble immune checkpoints with tissue-based biomarkers that are currently approved will be needed. While much work remains to be done, the studies reviewed here support the continued investigation of biomarkers in peripheral blood along with the analyses of tumor biopsies and suggest that there may be value in further exploring soluble immune checkpoints as potential biomarkers of clinical response, both in patients treated with ICI and conventional therapies.

### Supplementary Information

Additional file 1: Table S1. Changes in soluble immune checkpoints upon treatment with immune checkpoint therapy. Table S2. Baseline levels of other soluble immune checkpoints as indicators of clinical response to immune checkpoint therapy. Table S3. Post-treatment levels of other soluble immune checkpoints after immune checkpoint therapy as indicators of clinical response. Table S4. Changes in sPD-L1 upon treatment with conventional therapies. Table S5. Changes in sPD-1 and sCTLA4 upon treatment with conventional therapies. Table S6. Post-treatment levels of other soluble immune checkpoints after conventional therapies as indicators of clinical response. Table S7. Changes in sCD80, sTIM3, and sLAG3 upon treatment with conventional therapies. Table S8. Changes in sBTLA and sHVEM upon treatment with conventional therapies.

## Data Availability

Not applicable.

## Abbreviations

ICI: Immune checkpoint inhibitor
TMB: Tumor mutational burden
NLR: Neutrophil-to-lymphocyte ratio
APC: Antigen-presenting cell
ICOS: Inducible T cell co-stimulator
PD-1: Programmed cell death protein 1
PD-L1: Programmed cell death-ligand 1
PD-L2: Programmed cell death-ligand 2
CTLA4: Cytotoxic T-lymphocyte-associated antigen 4
TIM3: T cell immunoglobulin and mucin domain-containing protein 3
GAL9: Galectin 9
LAG3: Lymphocyte activation gene 3
BTLA: B and T lymphocyte attenuator
HVEM: Herpesvirus entry mediator
TIGIT: T cell immunoglobulin and ITIM domain
sPD-L1: Soluble programmed cell death-ligand 1
IFN-γ: Interferon gamma
IL-2: Interleukin 2
NSCLC: Non-small cell lung cancer
PFS: Progression-free survival
OS: Overall survival
ORR: Objective response rate
CR: Complete response
PR: Partial response
SD: Stable disease
sPD-1: Soluble programmed cell death protein 1
TNF-α: Tumor necrosis factor alpha
sCTLA4: Soluble cytotoxic T-lymphocyte-associated antigen 4
PBMC: Peripheral blood mononuclear cell
sCD80: Soluble CD80
TIL: Tumor-infiltrating lymphocyte
sTIM3: Soluble T cell immunoglobulin and mucin domain-containing protein 3
sLAG3: Soluble lymphocyte activation gene 3
sB7-H3: Soluble B7-H3
sBTLA: Soluble B and T lymphocyte attenuator
sHVEM: Soluble herpesvirus entry mediator
BOR: Best overall response
SCLC: Small cell lung cancer
DFS: Disease-free survival
HBV: Hepatitis B virus
TACE: Transarterial chemoembolization
PD: Progressive disease
ROC: Receiver operating characteristic
