# Supplementary material for: Soluble immune checkpoints: implications for cancer prognosis and response to immune checkpoint therapy and conventional therapies
Source: J Exp Clin Cancer Res. 2024 May 31;43:155. doi: 10.1186/s13046-024-03074-z (PMC11141022; doi:10.1186/s13046-024-03074-z)
Supplement: Supplementary file 1 — Additional file 1: Table S1. Changes in soluble immune checkpoints upon treatment with immune checkpoint therapy. Table S2. Baseline levels of other soluble immune checkpoints as indicators of clinical response to immune checkpoint therapy. Table S3. Post-treatment levels of other soluble immune checkpoints after immune checkpoint therapy as indicators of clinical response. Table S4. Changes in sPD-L1 upon treatment with conventional therapies. Table S5. Changes in sPD-1 and sCTLA4 upon treatment with conventional therapies. Table S6. Post-treatment levels of other soluble immune checkpoints after conventional therapies as indicators of clinical response. Table S7. Changes in sCD80, sTIM3, and sLAG3 upon treatment with conventional therapies. Table S8. Changes in sBTLA and sHVEM upon treatment with conventional therapies. [file 13046_2024_3074_MOESM1_ESM.pptx]

## Slide 1
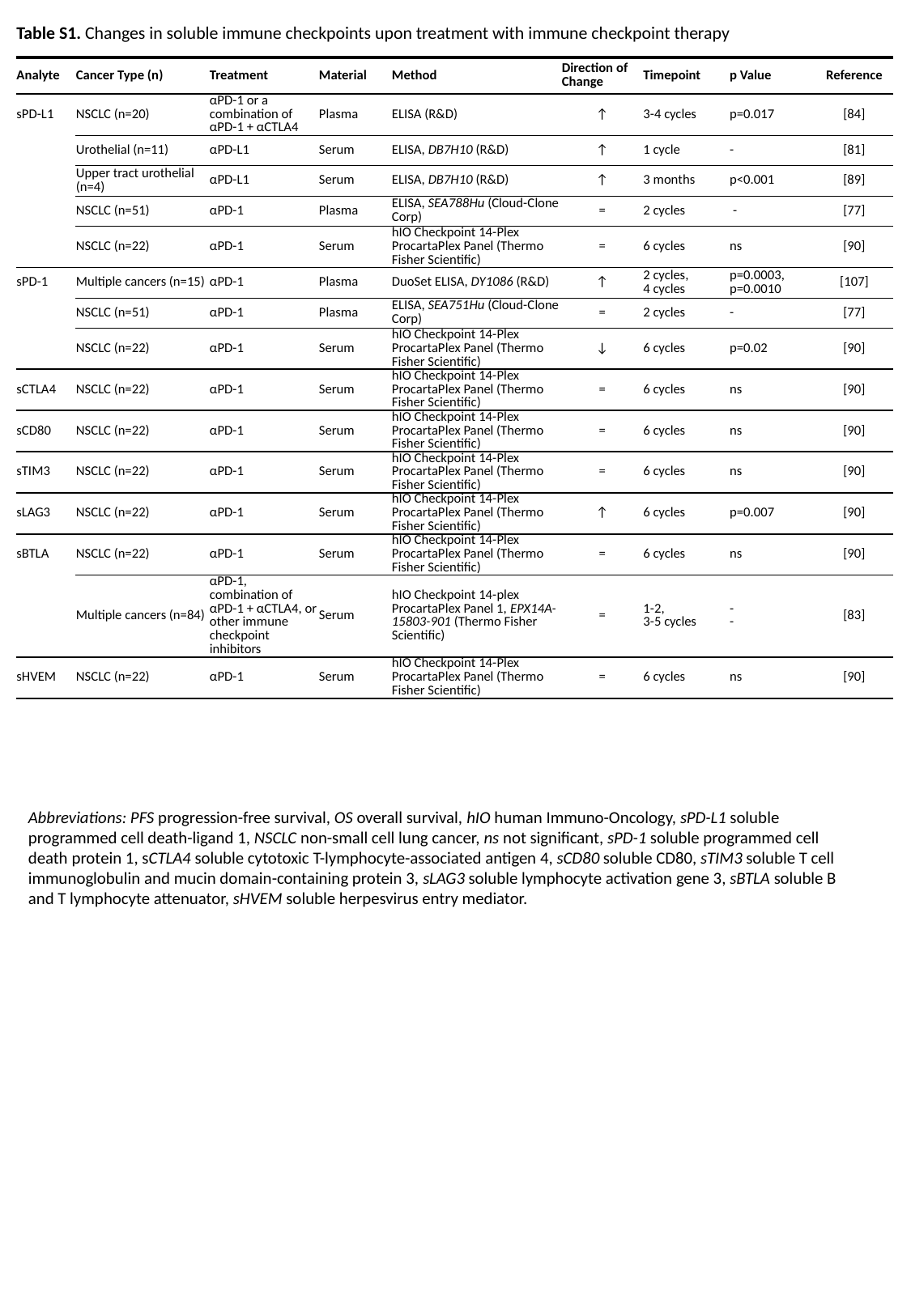

| Table S1. Changes in soluble immune checkpoints upon treatment with immune checkpoint therapy | | | | | | | | |
| --- | --- | --- | --- | --- | --- | --- | --- | --- |
| | | | | | | | | |
| Analyte | Cancer Type (n) | Treatment | Material | Method | Direction of Change | Timepoint | p Value | Reference |
| sPD-L1 | NSCLC (n=20) | αPD-1 or a combination of αPD-1 + αCTLA4 | Plasma | ELISA (R&D) | ↑ | 3-4 cycles | p=0.017 | [84] |
| | Urothelial (n=11) | αPD-L1 | Serum | ELISA, DB7H10 (R&D) | ↑ | 1 cycle | - | [81] |
| | Upper tract urothelial (n=4) | αPD-L1 | Serum | ELISA, DB7H10 (R&D) | ↑ | 3 months | p<0.001 | [89] |
| | NSCLC (n=51) | αPD-1 | Plasma | ELISA, SEA788Hu (Cloud-Clone Corp) | = | 2 cycles | - | [77] |
| | NSCLC (n=22) | αPD-1 | Serum | hIO Checkpoint 14-Plex ProcartaPlex Panel (Thermo Fisher Scientific) | = | 6 cycles | ns | [90] |
| sPD-1 | Multiple cancers (n=15) | αPD-1 | Plasma | DuoSet ELISA, DY1086 (R&D) | ↑ | 2 cycles, 4 cycles | p=0.0003, p=0.0010 | [107] |
| | NSCLC (n=51) | αPD-1 | Plasma | ELISA, SEA751Hu (Cloud-Clone Corp) | = | 2 cycles | - | [77] |
| | NSCLC (n=22) | αPD-1 | Serum | hIO Checkpoint 14-Plex ProcartaPlex Panel (Thermo Fisher Scientific) | ↓ | 6 cycles | p=0.02 | [90] |
| sCTLA4 | NSCLC (n=22) | αPD-1 | Serum | hIO Checkpoint 14-Plex ProcartaPlex Panel (Thermo Fisher Scientific) | = | 6 cycles | ns | [90] |
| sCD80 | NSCLC (n=22) | αPD-1 | Serum | hIO Checkpoint 14-Plex ProcartaPlex Panel (Thermo Fisher Scientific) | = | 6 cycles | ns | [90] |
| sTIM3 | NSCLC (n=22) | αPD-1 | Serum | hIO Checkpoint 14-Plex ProcartaPlex Panel (Thermo Fisher Scientific) | = | 6 cycles | ns | [90] |
| sLAG3 | NSCLC (n=22) | αPD-1 | Serum | hIO Checkpoint 14-Plex ProcartaPlex Panel (Thermo Fisher Scientific) | ↑ | 6 cycles | p=0.007 | [90] |
| sBTLA | NSCLC (n=22) | αPD-1 | Serum | hIO Checkpoint 14-Plex ProcartaPlex Panel (Thermo Fisher Scientific) | = | 6 cycles | ns | [90] |
| | Multiple cancers (n=84) | αPD-1, combination of αPD-1 + αCTLA4, or other immune checkpoint inhibitors | Serum | hIO Checkpoint 14-plex ProcartaPlex Panel 1, EPX14A-15803-901 (Thermo Fisher Scientific) | = | 1-2, 3-5 cycles | - - | [83] |
| sHVEM | NSCLC (n=22) | αPD-1 | Serum | hIO Checkpoint 14-Plex ProcartaPlex Panel (Thermo Fisher Scientific) | = | 6 cycles | ns | [90] |
Abbreviations: PFS progression-free survival, OS overall survival, hIO human Immuno-Oncology, sPD-L1 soluble programmed cell death-ligand 1, NSCLC non-small cell lung cancer, ns not significant, sPD-1 soluble programmed cell death protein 1, sCTLA4 soluble cytotoxic T-lymphocyte-associated antigen 4, sCD80 soluble CD80, sTIM3 soluble T cell immunoglobulin and mucin domain-containing protein 3, sLAG3 soluble lymphocyte activation gene 3, sBTLA soluble B and T lymphocyte attenuator, sHVEM soluble herpesvirus entry mediator.

## Slide 2
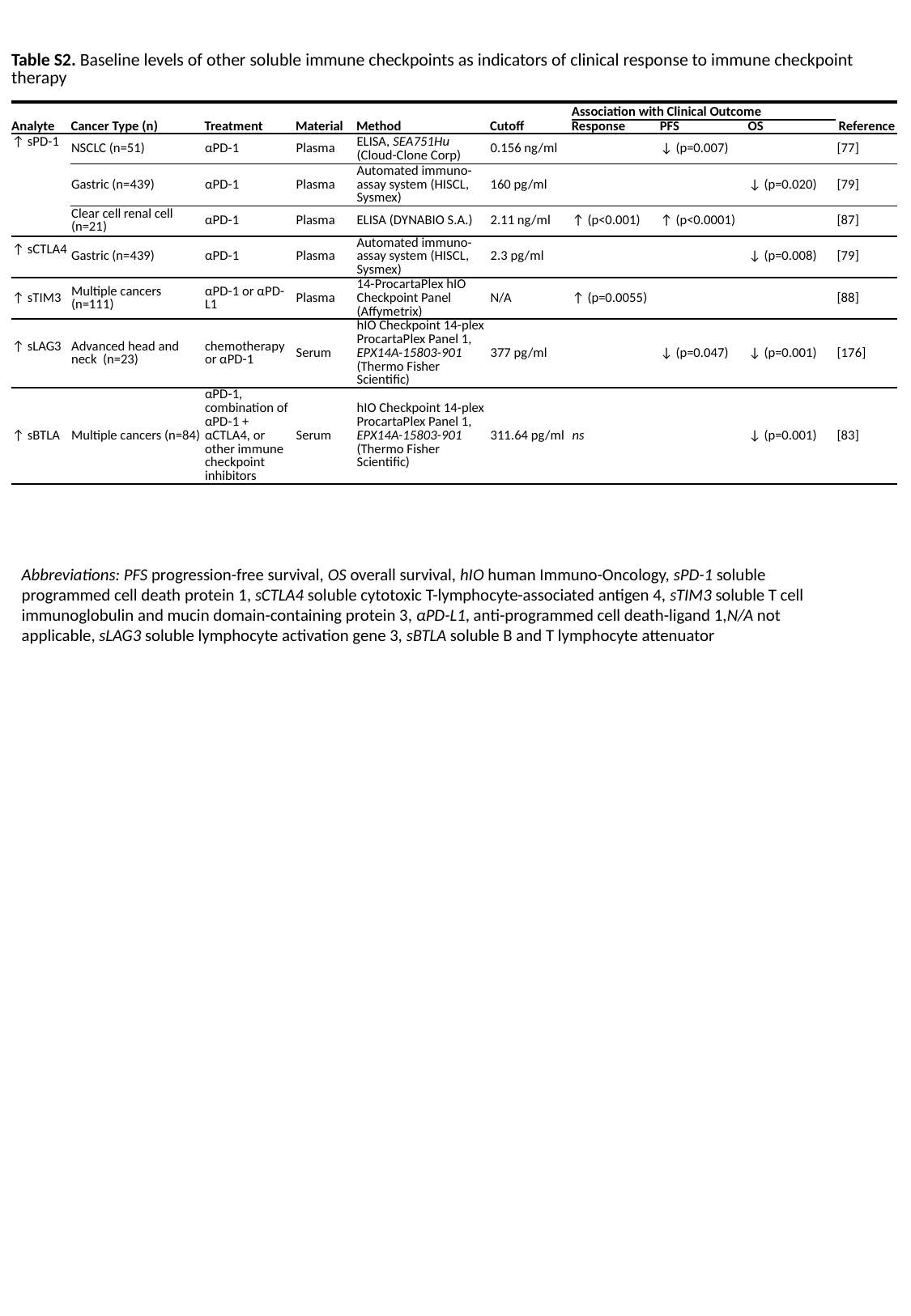

| Table S2. Baseline levels of other soluble immune checkpoints as indicators of clinical response to immune checkpoint therapy | | | | | | | | | |
| --- | --- | --- | --- | --- | --- | --- | --- | --- | --- |
| | | | | | | | | | |
| Analyte | | | | | | Association with Clinical Outcome | | | |
| | Cancer Type (n) | Treatment | Material | Method | Cutoff | Response | PFS | OS | Reference |
| ↑ sPD-1 | NSCLC (n=51) | αPD-1 | Plasma | ELISA, SEA751Hu (Cloud-Clone Corp) | 0.156 ng/ml | | ↓ (p=0.007) | | [77] |
| | Gastric (n=439) | αPD-1 | Plasma | Automated immuno-assay system (HISCL, Sysmex) | 160 pg/ml | | | ↓ (p=0.020) | [79] |
| | Clear cell renal cell (n=21) | αPD-1 | Plasma | ELISA (DYNABIO S.A.) | 2.11 ng/ml | ↑ (p<0.001) | ↑ (p<0.0001) | | [87] |
| ↑ sCTLA4 | Gastric (n=439) | αPD-1 | Plasma | Automated immuno-assay system (HISCL, Sysmex) | 2.3 pg/ml | | | ↓ (p=0.008) | [79] |
| ↑ sTIM3 | Multiple cancers (n=111) | αPD-1 or αPD-L1 | Plasma | 14-ProcartaPlex hIO Checkpoint Panel (Affymetrix) | N/A | ↑ (p=0.0055) | | | [88] |
| ↑ sLAG3 | Advanced head and neck (n=23) | chemotherapy or αPD-1 | Serum | hIO Checkpoint 14-plex ProcartaPlex Panel 1, EPX14A-15803-901 (Thermo Fisher Scientific) | 377 pg/ml | | ↓ (p=0.047) | ↓ (p=0.001) | [176] |
| ↑ sBTLA | Multiple cancers (n=84) | αPD-1, combination of αPD-1 + αCTLA4, or other immune checkpoint inhibitors | Serum | hIO Checkpoint 14-plex ProcartaPlex Panel 1, EPX14A-15803-901 (Thermo Fisher Scientific) | 311.64 pg/ml | ns | | ↓ (p=0.001) | [83] |
Abbreviations: PFS progression-free survival, OS overall survival, hIO human Immuno-Oncology, sPD-1 soluble programmed cell death protein 1, sCTLA4 soluble cytotoxic T-lymphocyte-associated antigen 4, sTIM3 soluble T cell immunoglobulin and mucin domain-containing protein 3, αPD-L1, anti-programmed cell death-ligand 1,N/A not applicable, sLAG3 soluble lymphocyte activation gene 3, sBTLA soluble B and T lymphocyte attenuator

## Slide 3
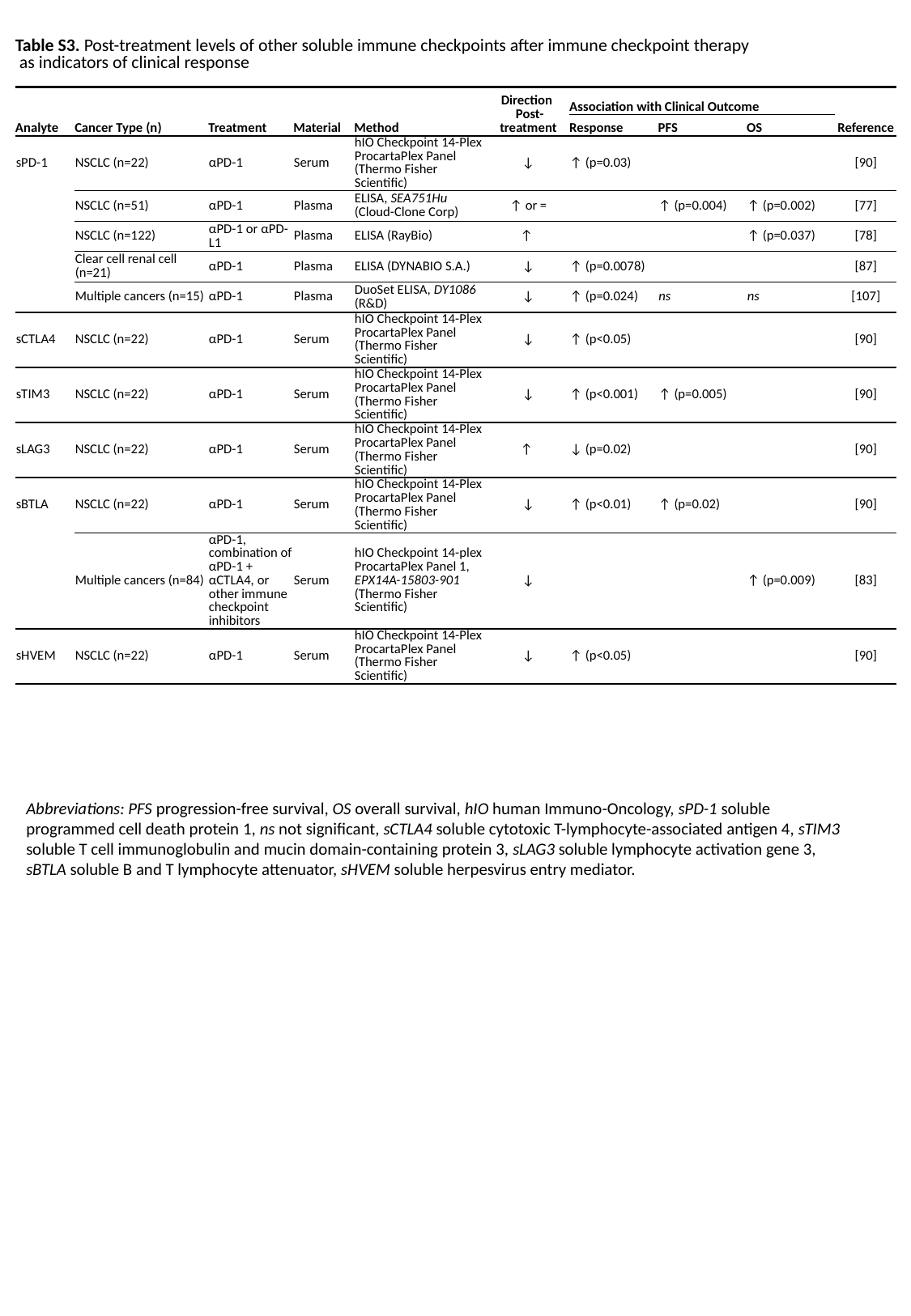

| Table S3. Post-treatment levels of other soluble immune checkpoints after immune checkpoint therapy as indicators of clinical response | | | | | | | | | |
| --- | --- | --- | --- | --- | --- | --- | --- | --- | --- |
| | | | | | | | | | |
| Analyte | Cancer Type (n) | Treatment | Material | Method | Direction Post-treatment | Association with Clinical Outcome | | | |
| Analyte | Cancer Type (n) | Treatment | Material | | Direction After Treatment | Response | PFS | OS | Reference |
| sPD-1 | NSCLC (n=22) | αPD-1 | Serum | hIO Checkpoint 14-Plex ProcartaPlex Panel (Thermo Fisher Scientific) | ↓ | ↑ (p=0.03) | | | [90] |
| | NSCLC (n=51) | αPD-1 | Plasma | ELISA, SEA751Hu (Cloud-Clone Corp) | ↑ or = | | ↑ (p=0.004) | ↑ (p=0.002) | [77] |
| | NSCLC (n=122) | αPD-1 or αPD-L1 | Plasma | ELISA (RayBio) | ↑ | | | ↑ (p=0.037) | [78] |
| | Clear cell renal cell (n=21) | αPD-1 | Plasma | ELISA (DYNABIO S.A.) | ↓ | ↑ (p=0.0078) | | | [87] |
| | Multiple cancers (n=15) | αPD-1 | Plasma | DuoSet ELISA, DY1086 (R&D) | ↓ | ↑ (p=0.024) | ns | ns | [107] |
| sCTLA4 | NSCLC (n=22) | αPD-1 | Serum | hIO Checkpoint 14-Plex ProcartaPlex Panel (Thermo Fisher Scientific) | ↓ | ↑ (p<0.05) | | | [90] |
| sTIM3 | NSCLC (n=22) | αPD-1 | Serum | hIO Checkpoint 14-Plex ProcartaPlex Panel (Thermo Fisher Scientific) | ↓ | ↑ (p<0.001) | ↑ (p=0.005) | | [90] |
| sLAG3 | NSCLC (n=22) | αPD-1 | Serum | hIO Checkpoint 14-Plex ProcartaPlex Panel (Thermo Fisher Scientific) | ↑ | ↓ (p=0.02) | | | [90] |
| sBTLA | NSCLC (n=22) | αPD-1 | Serum | hIO Checkpoint 14-Plex ProcartaPlex Panel (Thermo Fisher Scientific) | ↓ | ↑ (p<0.01) | ↑ (p=0.02) | | [90] |
| | Multiple cancers (n=84) | αPD-1, combination of αPD-1 + αCTLA4, or other immune checkpoint inhibitors | Serum | hIO Checkpoint 14-plex ProcartaPlex Panel 1, EPX14A-15803-901 (Thermo Fisher Scientific) | ↓ | | | ↑ (p=0.009) | [83] |
| sHVEM | NSCLC (n=22) | αPD-1 | Serum | hIO Checkpoint 14-Plex ProcartaPlex Panel (Thermo Fisher Scientific) | ↓ | ↑ (p<0.05) | | | [90] |
Abbreviations: PFS progression-free survival, OS overall survival, hIO human Immuno-Oncology, sPD-1 soluble programmed cell death protein 1, ns not significant, sCTLA4 soluble cytotoxic T-lymphocyte-associated antigen 4, sTIM3 soluble T cell immunoglobulin and mucin domain-containing protein 3, sLAG3 soluble lymphocyte activation gene 3, sBTLA soluble B and T lymphocyte attenuator, sHVEM soluble herpesvirus entry mediator.

## Slide 4
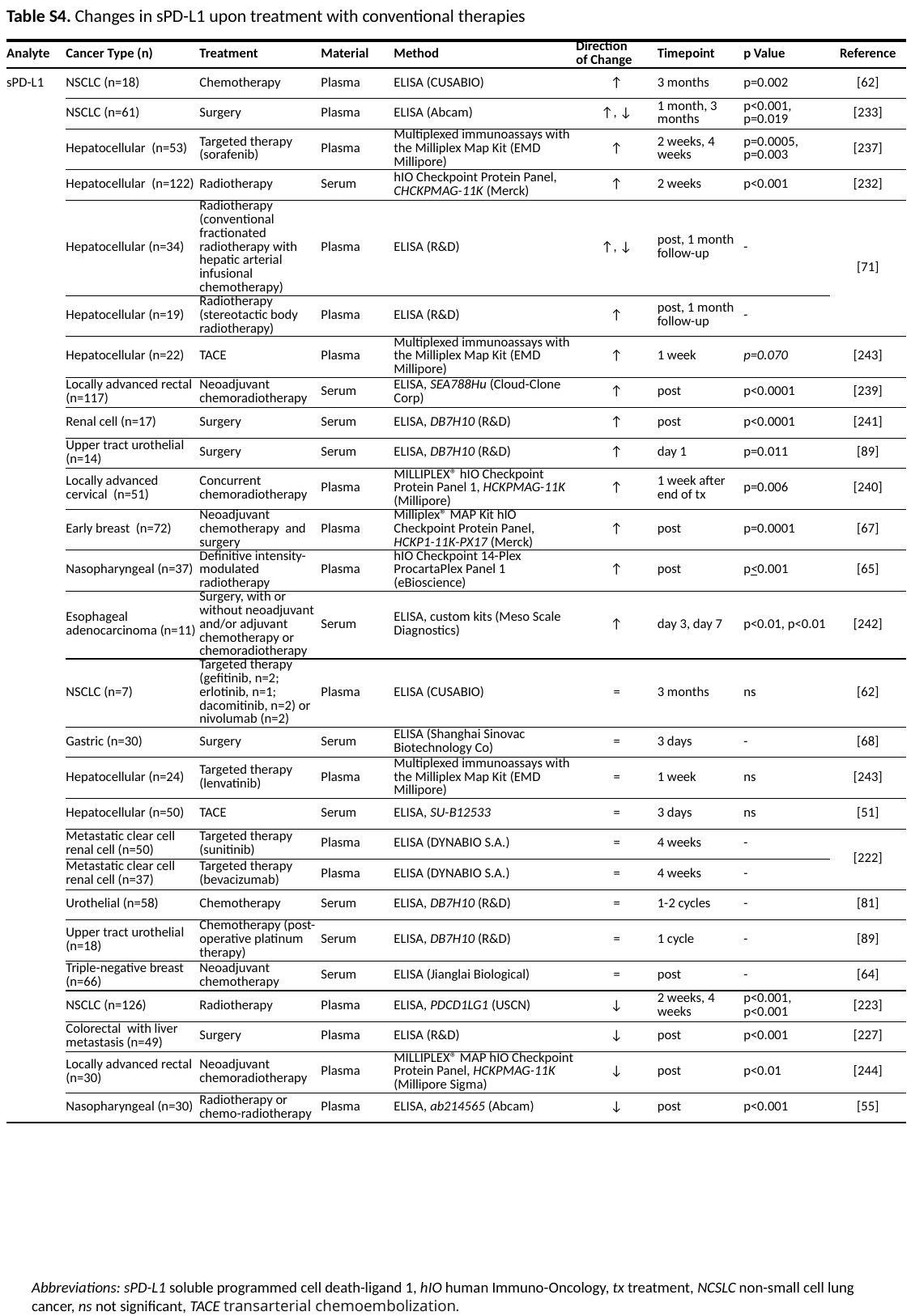

| Table S4. Changes in sPD-L1 upon treatment with conventional therapies | | | | | | | | |
| --- | --- | --- | --- | --- | --- | --- | --- | --- |
| | | | | | | | | |
| Analyte | Cancer Type (n) | Treatment | Material | Method | Direction of Change | Timepoint | p Value | Reference |
| sPD-L1 | NSCLC (n=18) | Chemotherapy | Plasma | ELISA (CUSABIO) | ↑ | 3 months | p=0.002 | [62] |
| | NSCLC (n=61) | Surgery | Plasma | ELISA (Abcam) | ↑, ↓ | 1 month, 3 months | p<0.001, p=0.019 | [233] |
| | Hepatocellular (n=53) | Targeted therapy (sorafenib) | Plasma | Multiplexed immunoassays with the Milliplex Map Kit (EMD Millipore) | ↑ | 2 weeks, 4 weeks | p=0.0005, p=0.003 | [237] |
| | Hepatocellular (n=122) | Radiotherapy | Serum | hIO Checkpoint Protein Panel, CHCKPMAG-11K (Merck) | ↑ | 2 weeks | p<0.001 | [232] |
| | Hepatocellular (n=34) | Radiotherapy (conventional fractionated radiotherapy with hepatic arterial infusional chemotherapy) | Plasma | ELISA (R&D) | ↑, ↓ | post, 1 month follow-up | - | [71] |
| | Hepatocellular (n=19) | Radiotherapy (stereotactic body radiotherapy) | Plasma | ELISA (R&D) | ↑ | post, 1 month follow-up | - | |
| | Hepatocellular (n=22) | TACE | Plasma | Multiplexed immunoassays with the Milliplex Map Kit (EMD Millipore) | ↑ | 1 week | p=0.070 | [243] |
| | Locally advanced rectal (n=117) | Neoadjuvant chemoradiotherapy | Serum | ELISA, SEA788Hu (Cloud-Clone Corp) | ↑ | post | p<0.0001 | [239] |
| | Renal cell (n=17) | Surgery | Serum | ELISA, DB7H10 (R&D) | ↑ | post | p<0.0001 | [241] |
| | Upper tract urothelial (n=14) | Surgery | Serum | ELISA, DB7H10 (R&D) | ↑ | day 1 | p=0.011 | [89] |
| | Locally advanced cervical (n=51) | Concurrent chemoradiotherapy | Plasma | MILLIPLEX® hIO Checkpoint Protein Panel 1, HCKPMAG-11K (Millipore) | ↑ | 1 week after end of tx | p=0.006 | [240] |
| | Early breast (n=72) | Neoadjuvant chemotherapy and surgery | Plasma | Milliplex® MAP Kit hIO Checkpoint Protein Panel, HCKP1-11K-PX17 (Merck) | ↑ | post | p=0.0001 | [67] |
| | Nasopharyngeal (n=37) | Definitive intensity-modulated radiotherapy | Plasma | hIO Checkpoint 14-Plex ProcartaPlex Panel 1 (eBioscience) | ↑ | post | p<0.001 | [65] |
| | Esophageal adenocarcinoma (n=11) | Surgery, with or without neoadjuvant and/or adjuvant chemotherapy or chemoradiotherapy | Serum | ELISA, custom kits (Meso Scale Diagnostics) | ↑ | day 3, day 7 | p<0.01, p<0.01 | [242] |
| | NSCLC (n=7) | Targeted therapy (gefitinib, n=2; erlotinib, n=1; dacomitinib, n=2) or nivolumab (n=2) | Plasma | ELISA (CUSABIO) | = | 3 months | ns | [62] |
| | Gastric (n=30) | Surgery | Serum | ELISA (Shanghai Sinovac Biotechnology Co) | = | 3 days | - | [68] |
| | Hepatocellular (n=24) | Targeted therapy (lenvatinib) | Plasma | Multiplexed immunoassays with the Milliplex Map Kit (EMD Millipore) | = | 1 week | ns | [243] |
| | Hepatocellular (n=50) | TACE | Serum | ELISA, SU-B12533 | = | 3 days | ns | [51] |
| | Metastatic clear cell renal cell (n=50) | Targeted therapy (sunitinib) | Plasma | ELISA (DYNABIO S.A.) | = | 4 weeks | - | [222] |
| | Metastatic clear cell renal cell (n=37) | Targeted therapy (bevacizumab) | Plasma | ELISA (DYNABIO S.A.) | = | 4 weeks | - | |
| | Urothelial (n=58) | Chemotherapy | Serum | ELISA, DB7H10 (R&D) | = | 1-2 cycles | - | [81] |
| | Upper tract urothelial (n=18) | Chemotherapy (post-operative platinum therapy) | Serum | ELISA, DB7H10 (R&D) | = | 1 cycle | - | [89] |
| | Triple-negative breast (n=66) | Neoadjuvant chemotherapy | Serum | ELISA (Jianglai Biological) | = | post | - | [64] |
| | NSCLC (n=126) | Radiotherapy | Plasma | ELISA, PDCD1LG1 (USCN) | ↓ | 2 weeks, 4 weeks | p<0.001, p<0.001 | [223] |
| | Colorectal with liver metastasis (n=49) | Surgery | Plasma | ELISA (R&D) | ↓ | post | p<0.001 | [227] |
| | Locally advanced rectal (n=30) | Neoadjuvant chemoradiotherapy | Plasma | MILLIPLEX® MAP hIO Checkpoint Protein Panel, HCKPMAG-11K (Millipore Sigma) | ↓ | post | p<0.01 | [244] |
| | Nasopharyngeal (n=30) | Radiotherapy or chemo-radiotherapy | Plasma | ELISA, ab214565 (Abcam) | ↓ | post | p<0.001 | [55] |
Abbreviations: sPD-L1 soluble programmed cell death-ligand 1, hIO human Immuno-Oncology, tx treatment, NCSLC non-small cell lung cancer, ns not significant, TACE transarterial chemoembolization.

## Slide 5
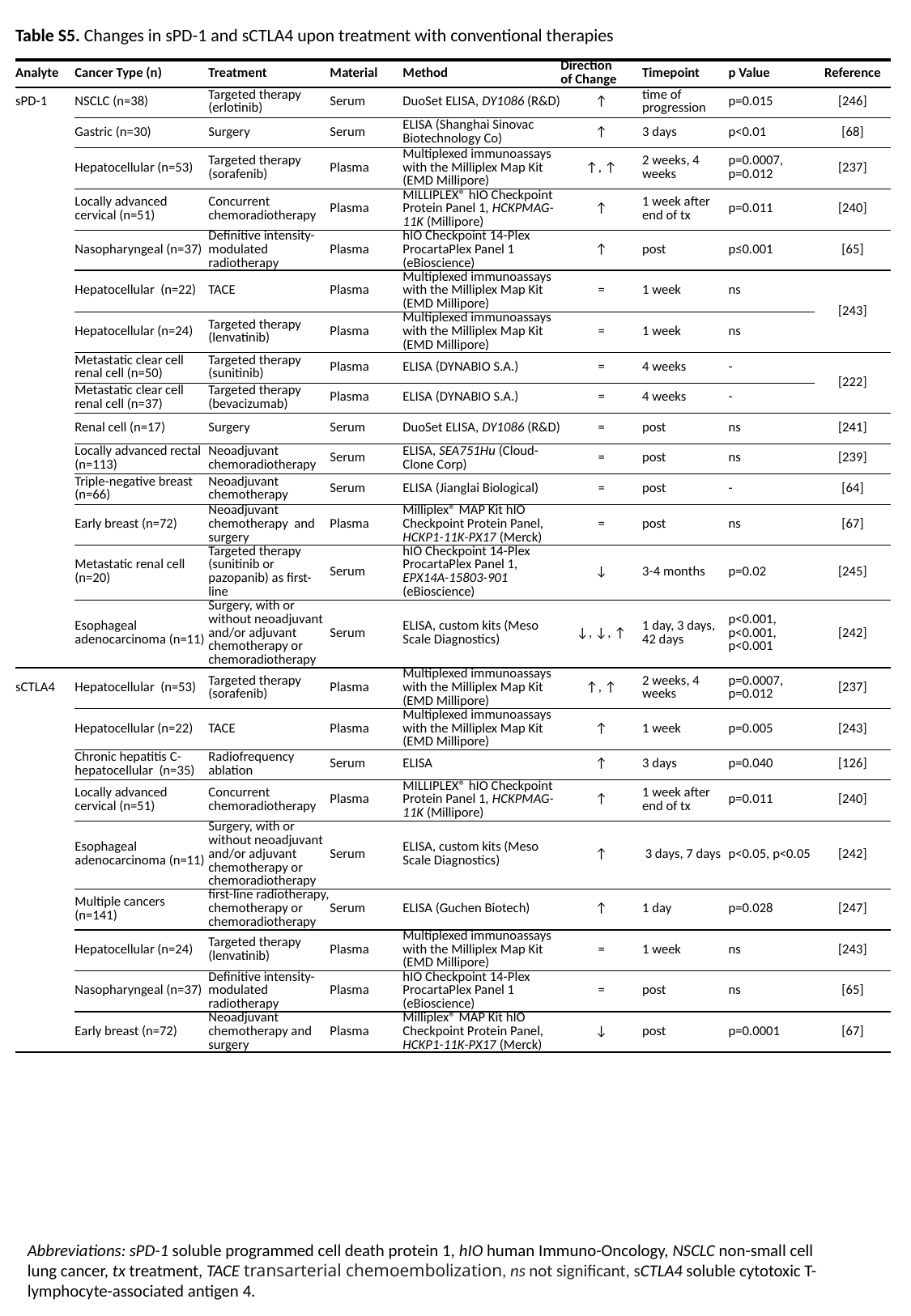

| Table S5. Changes in sPD-1 and sCTLA4 upon treatment with conventional therapies | | | | | | | | |
| --- | --- | --- | --- | --- | --- | --- | --- | --- |
| | | | | | | | | |
| Analyte | Cancer Type (n) | Treatment | Material | Method | Direction of Change | Timepoint | p Value | Reference |
| sPD-1 | NSCLC (n=38) | Targeted therapy (erlotinib) | Serum | DuoSet ELISA, DY1086 (R&D) | ↑ | time of progression | p=0.015 | [246] |
| | Gastric (n=30) | Surgery | Serum | ELISA (Shanghai Sinovac Biotechnology Co) | ↑ | 3 days | p<0.01 | [68] |
| | Hepatocellular (n=53) | Targeted therapy (sorafenib) | Plasma | Multiplexed immunoassays with the Milliplex Map Kit (EMD Millipore) | ↑, ↑ | 2 weeks, 4 weeks | p=0.0007, p=0.012 | [237] |
| | Locally advanced cervical (n=51) | Concurrent chemoradiotherapy | Plasma | MILLIPLEX® hIO Checkpoint Protein Panel 1, HCKPMAG-11K (Millipore) | ↑ | 1 week after end of tx | p=0.011 | [240] |
| | Nasopharyngeal (n=37) | Definitive intensity-modulated radiotherapy | Plasma | hIO Checkpoint 14-Plex ProcartaPlex Panel 1 (eBioscience) | ↑ | post | p≤0.001 | [65] |
| | Hepatocellular (n=22) | TACE | Plasma | Multiplexed immunoassays with the Milliplex Map Kit (EMD Millipore) | = | 1 week | ns | [243] |
| | Hepatocellular (n=24) | Targeted therapy (lenvatinib) | Plasma | Multiplexed immunoassays with the Milliplex Map Kit (EMD Millipore) | = | 1 week | ns | |
| | Metastatic clear cell renal cell (n=50) | Targeted therapy (sunitinib) | Plasma | ELISA (DYNABIO S.A.) | = | 4 weeks | - | [222] |
| | Metastatic clear cell renal cell (n=37) | Targeted therapy (bevacizumab) | Plasma | ELISA (DYNABIO S.A.) | = | 4 weeks | - | |
| | Renal cell (n=17) | Surgery | Serum | DuoSet ELISA, DY1086 (R&D) | = | post | ns | [241] |
| | Locally advanced rectal (n=113) | Neoadjuvant chemoradiotherapy | Serum | ELISA, SEA751Hu (Cloud-Clone Corp) | = | post | ns | [239] |
| | Triple-negative breast (n=66) | Neoadjuvant chemotherapy | Serum | ELISA (Jianglai Biological) | = | post | - | [64] |
| | Early breast (n=72) | Neoadjuvant chemotherapy and surgery | Plasma | Milliplex® MAP Kit hIO Checkpoint Protein Panel, HCKP1-11K-PX17 (Merck) | = | post | ns | [67] |
| | Metastatic renal cell (n=20) | Targeted therapy (sunitinib or pazopanib) as first-line | Serum | hIO Checkpoint 14-Plex ProcartaPlex Panel 1, EPX14A-15803-901 (eBioscience) | ↓ | 3-4 months | p=0.02 | [245] |
| | Esophageal adenocarcinoma (n=11) | Surgery, with or without neoadjuvant and/or adjuvant chemotherapy or chemoradiotherapy | Serum | ELISA, custom kits (Meso Scale Diagnostics) | ↓, ↓, ↑ | 1 day, 3 days, 42 days | p<0.001, p<0.001, p<0.001 | [242] |
| sCTLA4 | Hepatocellular (n=53) | Targeted therapy (sorafenib) | Plasma | Multiplexed immunoassays with the Milliplex Map Kit (EMD Millipore) | ↑, ↑ | 2 weeks, 4 weeks | p=0.0007, p=0.012 | [237] |
| | Hepatocellular (n=22) | TACE | Plasma | Multiplexed immunoassays with the Milliplex Map Kit (EMD Millipore) | ↑ | 1 week | p=0.005 | [243] |
| | Chronic hepatitis C-hepatocellular (n=35) | Radiofrequency ablation | Serum | ELISA | ↑ | 3 days | p=0.040 | [126] |
| | Locally advanced cervical (n=51) | Concurrent chemoradiotherapy | Plasma | MILLIPLEX® hIO Checkpoint Protein Panel 1, HCKPMAG-11K (Millipore) | ↑ | 1 week after end of tx | p=0.011 | [240] |
| | Esophageal adenocarcinoma (n=11) | Surgery, with or without neoadjuvant and/or adjuvant chemotherapy or chemoradiotherapy | Serum | ELISA, custom kits (Meso Scale Diagnostics) | ↑ | 3 days, 7 days | p<0.05, p<0.05 | [242] |
| | Multiple cancers (n=141) | first-line radiotherapy, chemotherapy or chemoradiotherapy | Serum | ELISA (Guchen Biotech) | ↑ | 1 day | p=0.028 | [247] |
| | Hepatocellular (n=24) | Targeted therapy (lenvatinib) | Plasma | Multiplexed immunoassays with the Milliplex Map Kit (EMD Millipore) | = | 1 week | ns | [243] |
| | Nasopharyngeal (n=37) | Definitive intensity-modulated radiotherapy | Plasma | hIO Checkpoint 14-Plex ProcartaPlex Panel 1 (eBioscience) | = | post | ns | [65] |
| | Early breast (n=72) | Neoadjuvant chemotherapy and surgery | Plasma | Milliplex® MAP Kit hIO Checkpoint Protein Panel, HCKP1-11K-PX17 (Merck) | ↓ | post | p=0.0001 | [67] |
Abbreviations: sPD-1 soluble programmed cell death protein 1, hIO human Immuno-Oncology, NSCLC non-small cell lung cancer, tx treatment, TACE transarterial chemoembolization, ns not significant, sCTLA4 soluble cytotoxic T-lymphocyte-associated antigen 4.

## Slide 6
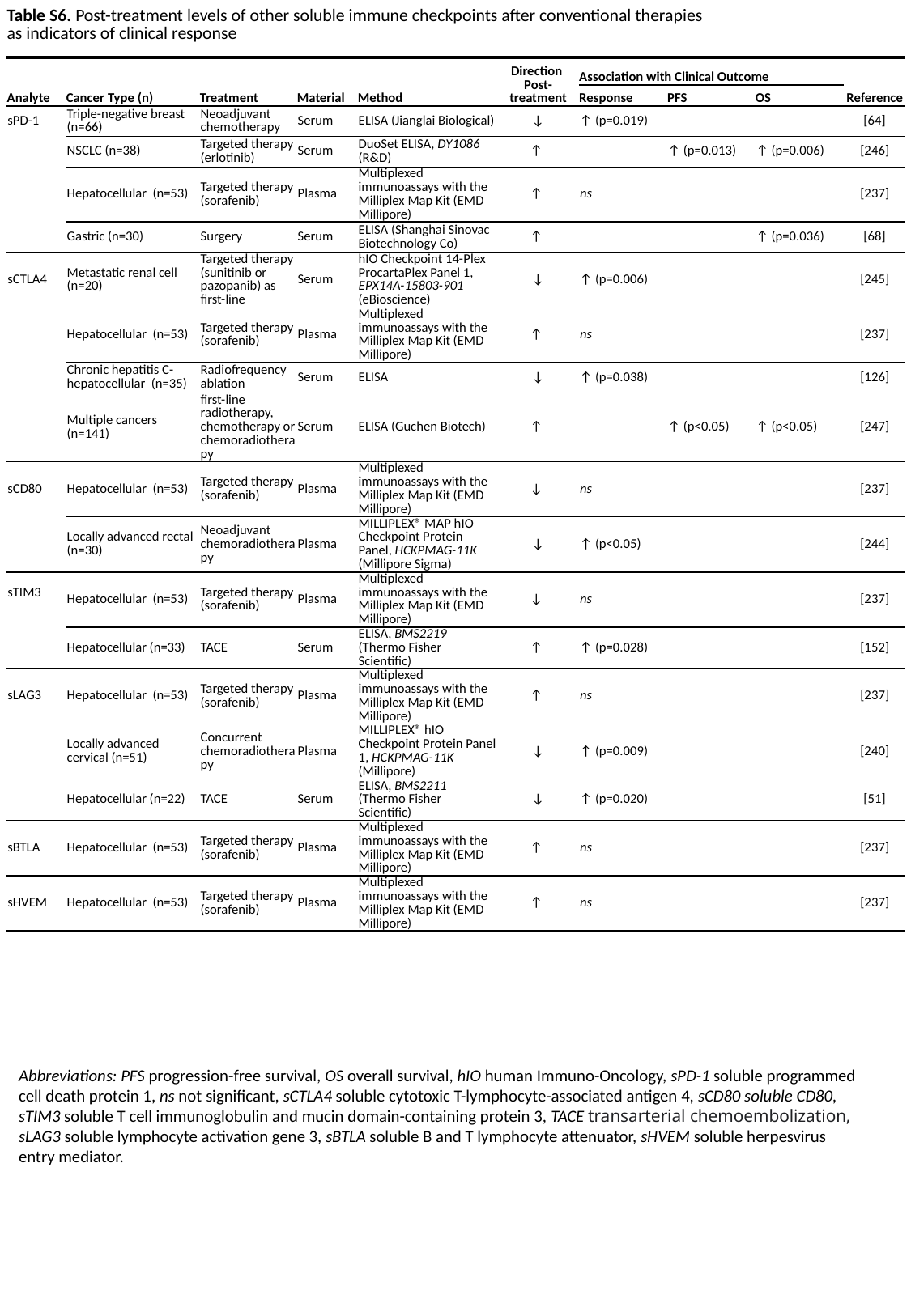

| Table S6. Post-treatment levels of other soluble immune checkpoints after conventional therapies as indicators of clinical response | | | | | | | | | |
| --- | --- | --- | --- | --- | --- | --- | --- | --- | --- |
| | | | | | | | | | |
| Analyte | Cancer Type (n) | Treatment | Material | Method | Direction Post-treatment | Association with Clinical Outcome | | | |
| Analyte | Cancer Type (n) | Treatment | Material | | Direction After Treatment | Response | PFS | OS | Reference |
| sPD-1 | Triple-negative breast (n=66) | Neoadjuvant chemotherapy | Serum | ELISA (Jianglai Biological) | ↓ | ↑ (p=0.019) | | | [64] |
| | NSCLC (n=38) | Targeted therapy (erlotinib) | Serum | DuoSet ELISA, DY1086 (R&D) | ↑ | | ↑ (p=0.013) | ↑ (p=0.006) | [246] |
| | Hepatocellular (n=53) | Targeted therapy (sorafenib) | Plasma | Multiplexed immunoassays with the Milliplex Map Kit (EMD Millipore) | ↑ | ns | | | [237] |
| | Gastric (n=30) | Surgery | Serum | ELISA (Shanghai Sinovac Biotechnology Co) | ↑ | | | ↑ (p=0.036) | [68] |
| sCTLA4 | Metastatic renal cell (n=20) | Targeted therapy (sunitinib or pazopanib) as first-line | Serum | hIO Checkpoint 14-Plex ProcartaPlex Panel 1, EPX14A-15803-901 (eBioscience) | ↓ | ↑ (p=0.006) | | | [245] |
| | Hepatocellular (n=53) | Targeted therapy (sorafenib) | Plasma | Multiplexed immunoassays with the Milliplex Map Kit (EMD Millipore) | ↑ | ns | | | [237] |
| | Chronic hepatitis C-hepatocellular (n=35) | Radiofrequency ablation | Serum | ELISA | ↓ | ↑ (p=0.038) | | | [126] |
| | Multiple cancers (n=141) | first-line radiotherapy, chemotherapy or chemoradiotherapy | Serum | ELISA (Guchen Biotech) | ↑ | | ↑ (p<0.05) | ↑ (p<0.05) | [247] |
| sCD80 | Hepatocellular (n=53) | Targeted therapy (sorafenib) | Plasma | Multiplexed immunoassays with the Milliplex Map Kit (EMD Millipore) | ↓ | ns | | | [237] |
| | Locally advanced rectal (n=30) | Neoadjuvant chemoradiotherapy | Plasma | MILLIPLEX® MAP hIO Checkpoint Protein Panel, HCKPMAG-11K (Millipore Sigma) | ↓ | ↑ (p<0.05) | | | [244] |
| sTIM3 | Hepatocellular (n=53) | Targeted therapy (sorafenib) | Plasma | Multiplexed immunoassays with the Milliplex Map Kit (EMD Millipore) | ↓ | ns | | | [237] |
| | Hepatocellular (n=33) | TACE | Serum | ELISA, BMS2219 (Thermo Fisher Scientific) | ↑ | ↑ (p=0.028) | | | [152] |
| sLAG3 | Hepatocellular (n=53) | Targeted therapy (sorafenib) | Plasma | Multiplexed immunoassays with the Milliplex Map Kit (EMD Millipore) | ↑ | ns | | | [237] |
| | Locally advanced cervical (n=51) | Concurrent chemoradiotherapy | Plasma | MILLIPLEX® hIO Checkpoint Protein Panel 1, HCKPMAG-11K (Millipore) | ↓ | ↑ (p=0.009) | | | [240] |
| | Hepatocellular (n=22) | TACE | Serum | ELISA, BMS2211 (Thermo Fisher Scientific) | ↓ | ↑ (p=0.020) | | | [51] |
| sBTLA | Hepatocellular (n=53) | Targeted therapy (sorafenib) | Plasma | Multiplexed immunoassays with the Milliplex Map Kit (EMD Millipore) | ↑ | ns | | | [237] |
| sHVEM | Hepatocellular (n=53) | Targeted therapy (sorafenib) | Plasma | Multiplexed immunoassays with the Milliplex Map Kit (EMD Millipore) | ↑ | ns | | | [237] |
Abbreviations: PFS progression-free survival, OS overall survival, hIO human Immuno-Oncology, sPD-1 soluble programmed cell death protein 1, ns not significant, sCTLA4 soluble cytotoxic T-lymphocyte-associated antigen 4, sCD80 soluble CD80, sTIM3 soluble T cell immunoglobulin and mucin domain-containing protein 3, TACE transarterial chemoembolization, sLAG3 soluble lymphocyte activation gene 3, sBTLA soluble B and T lymphocyte attenuator, sHVEM soluble herpesvirus entry mediator.

## Slide 7
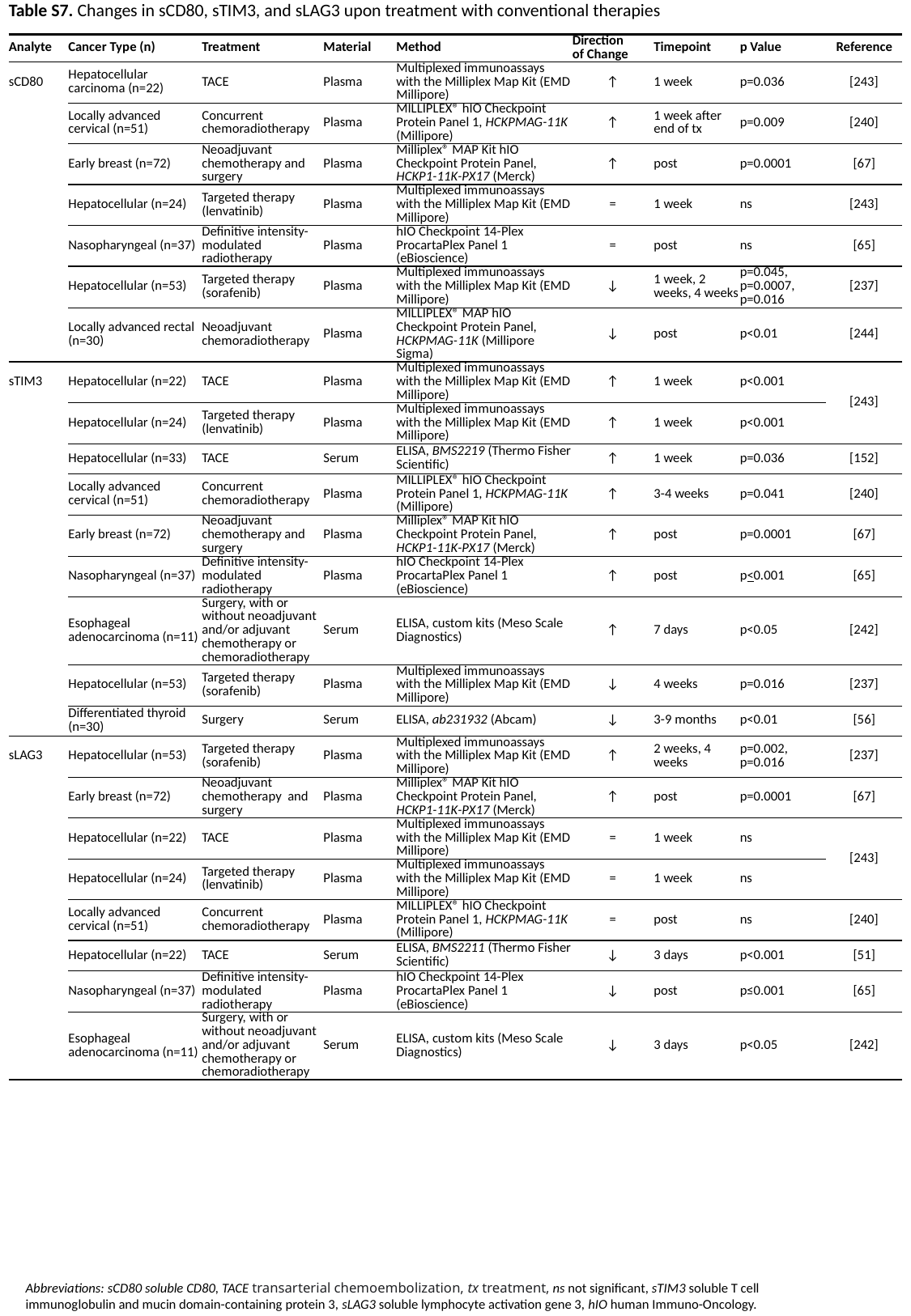

| Table S7. Changes in sCD80, sTIM3, and sLAG3 upon treatment with conventional therapies | | | | | | | | |
| --- | --- | --- | --- | --- | --- | --- | --- | --- |
| | | | | | | | | |
| Analyte | Cancer Type (n) | Treatment | Material | Method | Direction of Change | Timepoint | p Value | Reference |
| sCD80 | Hepatocellular carcinoma (n=22) | TACE | Plasma | Multiplexed immunoassays with the Milliplex Map Kit (EMD Millipore) | ↑ | 1 week | p=0.036 | [243] |
| | Locally advanced cervical (n=51) | Concurrent chemoradiotherapy | Plasma | MILLIPLEX® hIO Checkpoint Protein Panel 1, HCKPMAG-11K (Millipore) | ↑ | 1 week after end of tx | p=0.009 | [240] |
| | Early breast (n=72) | Neoadjuvant chemotherapy and surgery | Plasma | Milliplex® MAP Kit hIO Checkpoint Protein Panel, HCKP1-11K-PX17 (Merck) | ↑ | post | p=0.0001 | [67] |
| | Hepatocellular (n=24) | Targeted therapy (lenvatinib) | Plasma | Multiplexed immunoassays with the Milliplex Map Kit (EMD Millipore) | = | 1 week | ns | [243] |
| | Nasopharyngeal (n=37) | Definitive intensity-modulated radiotherapy | Plasma | hIO Checkpoint 14-Plex ProcartaPlex Panel 1 (eBioscience) | = | post | ns | [65] |
| | Hepatocellular (n=53) | Targeted therapy (sorafenib) | Plasma | Multiplexed immunoassays with the Milliplex Map Kit (EMD Millipore) | ↓ | 1 week, 2 weeks, 4 weeks | p=0.045, p=0.0007, p=0.016 | [237] |
| | Locally advanced rectal (n=30) | Neoadjuvant chemoradiotherapy | Plasma | MILLIPLEX® MAP hIO Checkpoint Protein Panel, HCKPMAG-11K (Millipore Sigma) | ↓ | post | p<0.01 | [244] |
| sTIM3 | Hepatocellular (n=22) | TACE | Plasma | Multiplexed immunoassays with the Milliplex Map Kit (EMD Millipore) | ↑ | 1 week | p<0.001 | [243] |
| | Hepatocellular (n=24) | Targeted therapy (lenvatinib) | Plasma | Multiplexed immunoassays with the Milliplex Map Kit (EMD Millipore) | ↑ | 1 week | p<0.001 | |
| | Hepatocellular (n=33) | TACE | Serum | ELISA, BMS2219 (Thermo Fisher Scientific) | ↑ | 1 week | p=0.036 | [152] |
| | Locally advanced cervical (n=51) | Concurrent chemoradiotherapy | Plasma | MILLIPLEX® hIO Checkpoint Protein Panel 1, HCKPMAG-11K (Millipore) | ↑ | 3-4 weeks | p=0.041 | [240] |
| | Early breast (n=72) | Neoadjuvant chemotherapy and surgery | Plasma | Milliplex® MAP Kit hIO Checkpoint Protein Panel, HCKP1-11K-PX17 (Merck) | ↑ | post | p=0.0001 | [67] |
| | Nasopharyngeal (n=37) | Definitive intensity-modulated radiotherapy | Plasma | hIO Checkpoint 14-Plex ProcartaPlex Panel 1 (eBioscience) | ↑ | post | p<0.001 | [65] |
| | Esophageal adenocarcinoma (n=11) | Surgery, with or without neoadjuvant and/or adjuvant chemotherapy or chemoradiotherapy | Serum | ELISA, custom kits (Meso Scale Diagnostics) | ↑ | 7 days | p<0.05 | [242] |
| | Hepatocellular (n=53) | Targeted therapy (sorafenib) | Plasma | Multiplexed immunoassays with the Milliplex Map Kit (EMD Millipore) | ↓ | 4 weeks | p=0.016 | [237] |
| | Differentiated thyroid (n=30) | Surgery | Serum | ELISA, ab231932 (Abcam) | ↓ | 3-9 months | p<0.01 | [56] |
| sLAG3 | Hepatocellular (n=53) | Targeted therapy (sorafenib) | Plasma | Multiplexed immunoassays with the Milliplex Map Kit (EMD Millipore) | ↑ | 2 weeks, 4 weeks | p=0.002, p=0.016 | [237] |
| | Early breast (n=72) | Neoadjuvant chemotherapy and surgery | Plasma | Milliplex® MAP Kit hIO Checkpoint Protein Panel, HCKP1-11K-PX17 (Merck) | ↑ | post | p=0.0001 | [67] |
| | Hepatocellular (n=22) | TACE | Plasma | Multiplexed immunoassays with the Milliplex Map Kit (EMD Millipore) | = | 1 week | ns | [243] |
| | Hepatocellular (n=24) | Targeted therapy (lenvatinib) | Plasma | Multiplexed immunoassays with the Milliplex Map Kit (EMD Millipore) | = | 1 week | ns | |
| | Locally advanced cervical (n=51) | Concurrent chemoradiotherapy | Plasma | MILLIPLEX® hIO Checkpoint Protein Panel 1, HCKPMAG-11K (Millipore) | = | post | ns | [240] |
| | Hepatocellular (n=22) | TACE | Serum | ELISA, BMS2211 (Thermo Fisher Scientific) | ↓ | 3 days | p<0.001 | [51] |
| | Nasopharyngeal (n=37) | Definitive intensity-modulated radiotherapy | Plasma | hIO Checkpoint 14-Plex ProcartaPlex Panel 1 (eBioscience) | ↓ | post | p≤0.001 | [65] |
| | Esophageal adenocarcinoma (n=11) | Surgery, with or without neoadjuvant and/or adjuvant chemotherapy or chemoradiotherapy | Serum | ELISA, custom kits (Meso Scale Diagnostics) | ↓ | 3 days | p<0.05 | [242] |
Abbreviations: sCD80 soluble CD80, TACE transarterial chemoembolization, tx treatment, ns not significant, sTIM3 soluble T cell immunoglobulin and mucin domain-containing protein 3, sLAG3 soluble lymphocyte activation gene 3, hIO human Immuno-Oncology.

## Slide 8
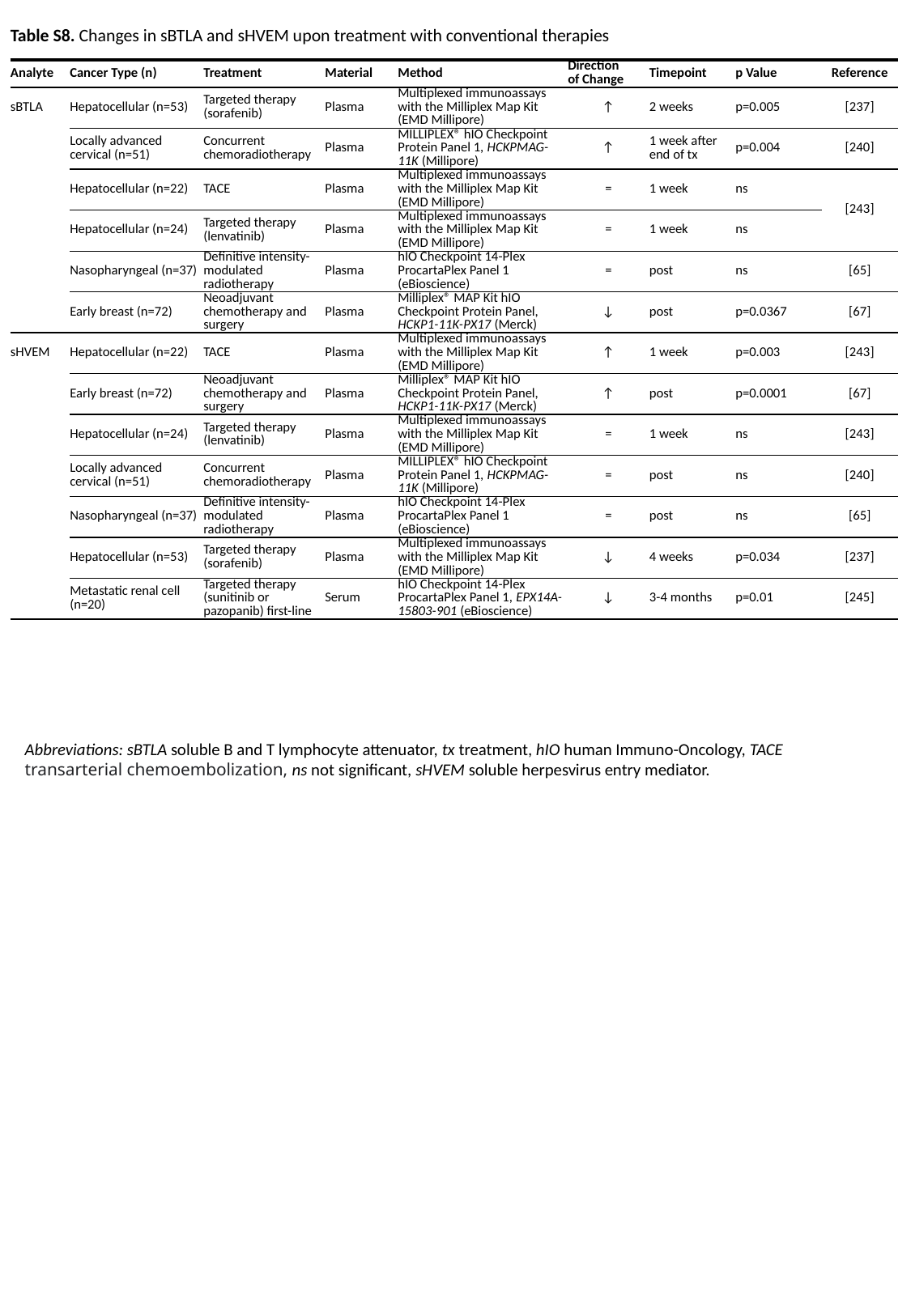

| Table S8. Changes in sBTLA and sHVEM upon treatment with conventional therapies | | | | | | | | |
| --- | --- | --- | --- | --- | --- | --- | --- | --- |
| | | | | | | | | |
| Analyte | Cancer Type (n) | Treatment | Material | Method | Direction of Change | Timepoint | p Value | Reference |
| sBTLA | Hepatocellular (n=53) | Targeted therapy (sorafenib) | Plasma | Multiplexed immunoassays with the Milliplex Map Kit (EMD Millipore) | ↑ | 2 weeks | p=0.005 | [237] |
| | Locally advanced cervical (n=51) | Concurrent chemoradiotherapy | Plasma | MILLIPLEX® hIO Checkpoint Protein Panel 1, HCKPMAG-11K (Millipore) | ↑ | 1 week after end of tx | p=0.004 | [240] |
| | Hepatocellular (n=22) | TACE | Plasma | Multiplexed immunoassays with the Milliplex Map Kit (EMD Millipore) | = | 1 week | ns | [243] |
| | Hepatocellular (n=24) | Targeted therapy (lenvatinib) | Plasma | Multiplexed immunoassays with the Milliplex Map Kit (EMD Millipore) | = | 1 week | ns | |
| | Nasopharyngeal (n=37) | Definitive intensity-modulated radiotherapy | Plasma | hIO Checkpoint 14-Plex ProcartaPlex Panel 1 (eBioscience) | = | post | ns | [65] |
| | Early breast (n=72) | Neoadjuvant chemotherapy and surgery | Plasma | Milliplex® MAP Kit hIO Checkpoint Protein Panel, HCKP1-11K-PX17 (Merck) | ↓ | post | p=0.0367 | [67] |
| sHVEM | Hepatocellular (n=22) | TACE | Plasma | Multiplexed immunoassays with the Milliplex Map Kit (EMD Millipore) | ↑ | 1 week | p=0.003 | [243] |
| | Early breast (n=72) | Neoadjuvant chemotherapy and surgery | Plasma | Milliplex® MAP Kit hIO Checkpoint Protein Panel, HCKP1-11K-PX17 (Merck) | ↑ | post | p=0.0001 | [67] |
| | Hepatocellular (n=24) | Targeted therapy (lenvatinib) | Plasma | Multiplexed immunoassays with the Milliplex Map Kit (EMD Millipore) | = | 1 week | ns | [243] |
| | Locally advanced cervical (n=51) | Concurrent chemoradiotherapy | Plasma | MILLIPLEX® hIO Checkpoint Protein Panel 1, HCKPMAG-11K (Millipore) | = | post | ns | [240] |
| | Nasopharyngeal (n=37) | Definitive intensity-modulated radiotherapy | Plasma | hIO Checkpoint 14-Plex ProcartaPlex Panel 1 (eBioscience) | = | post | ns | [65] |
| | Hepatocellular (n=53) | Targeted therapy (sorafenib) | Plasma | Multiplexed immunoassays with the Milliplex Map Kit (EMD Millipore) | ↓ | 4 weeks | p=0.034 | [237] |
| | Metastatic renal cell (n=20) | Targeted therapy (sunitinib or pazopanib) first-line | Serum | hIO Checkpoint 14-Plex ProcartaPlex Panel 1, EPX14A-15803-901 (eBioscience) | ↓ | 3-4 months | p=0.01 | [245] |
Abbreviations: sBTLA soluble B and T lymphocyte attenuator, tx treatment, hIO human Immuno-Oncology, TACE transarterial chemoembolization, ns not significant, sHVEM soluble herpesvirus entry mediator.
